# Supplementary figures and images for: Rapid cell type-specific nascent proteome labeling in Drosophila
Source: eLife. 2023 Apr 24;12:e83545. doi: 10.7554/eLife.83545 (PMC10125018; doi:10.7554/eLife.83545)

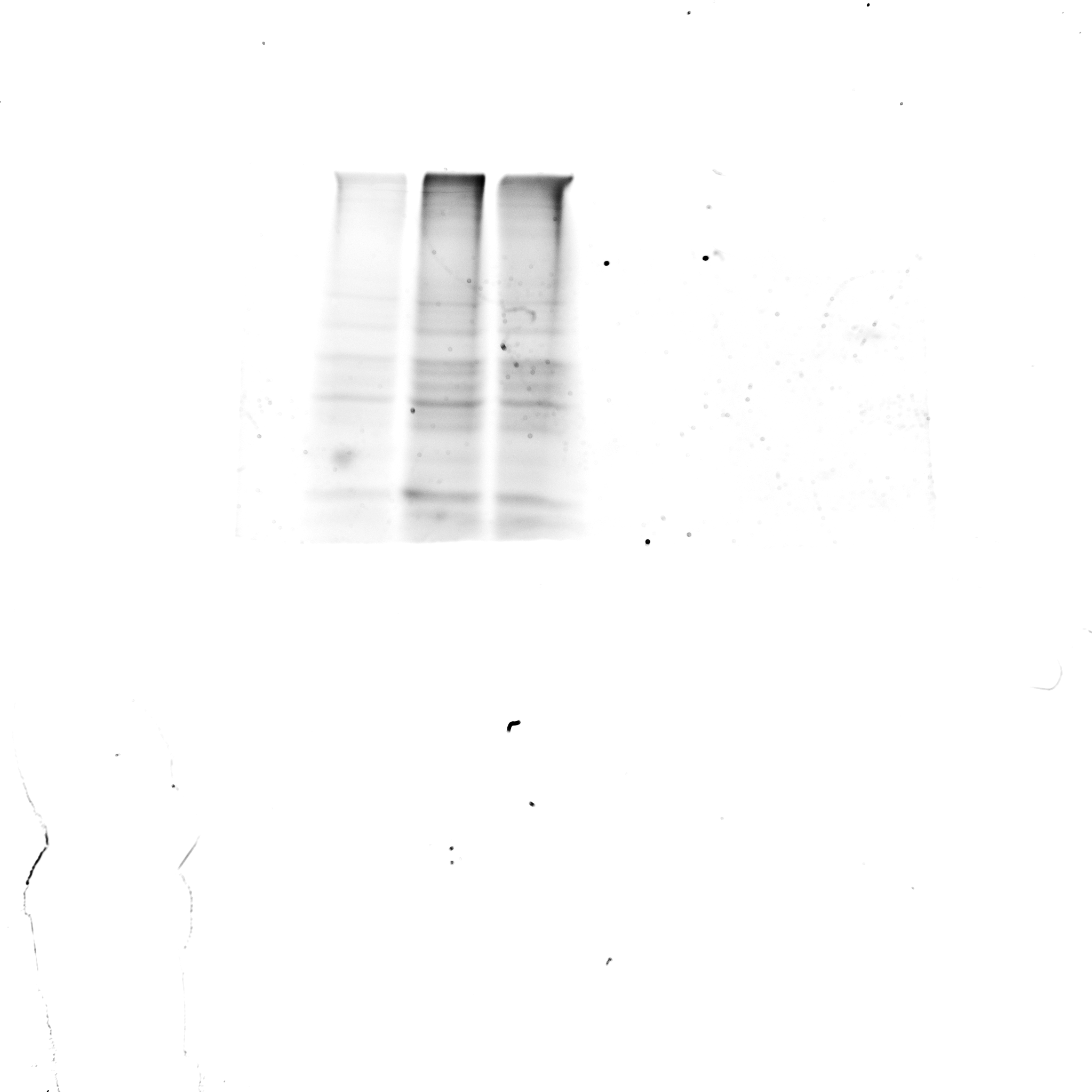

Supplement: Figure 1—source data 1. [file elife-83545-fig1-data1.zip › Figure 1/Fig_1E_fluorescence_original.tiff]

OPP

-

+

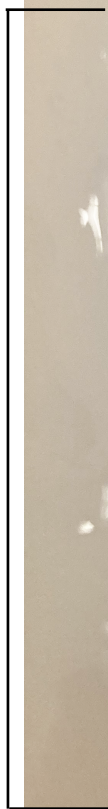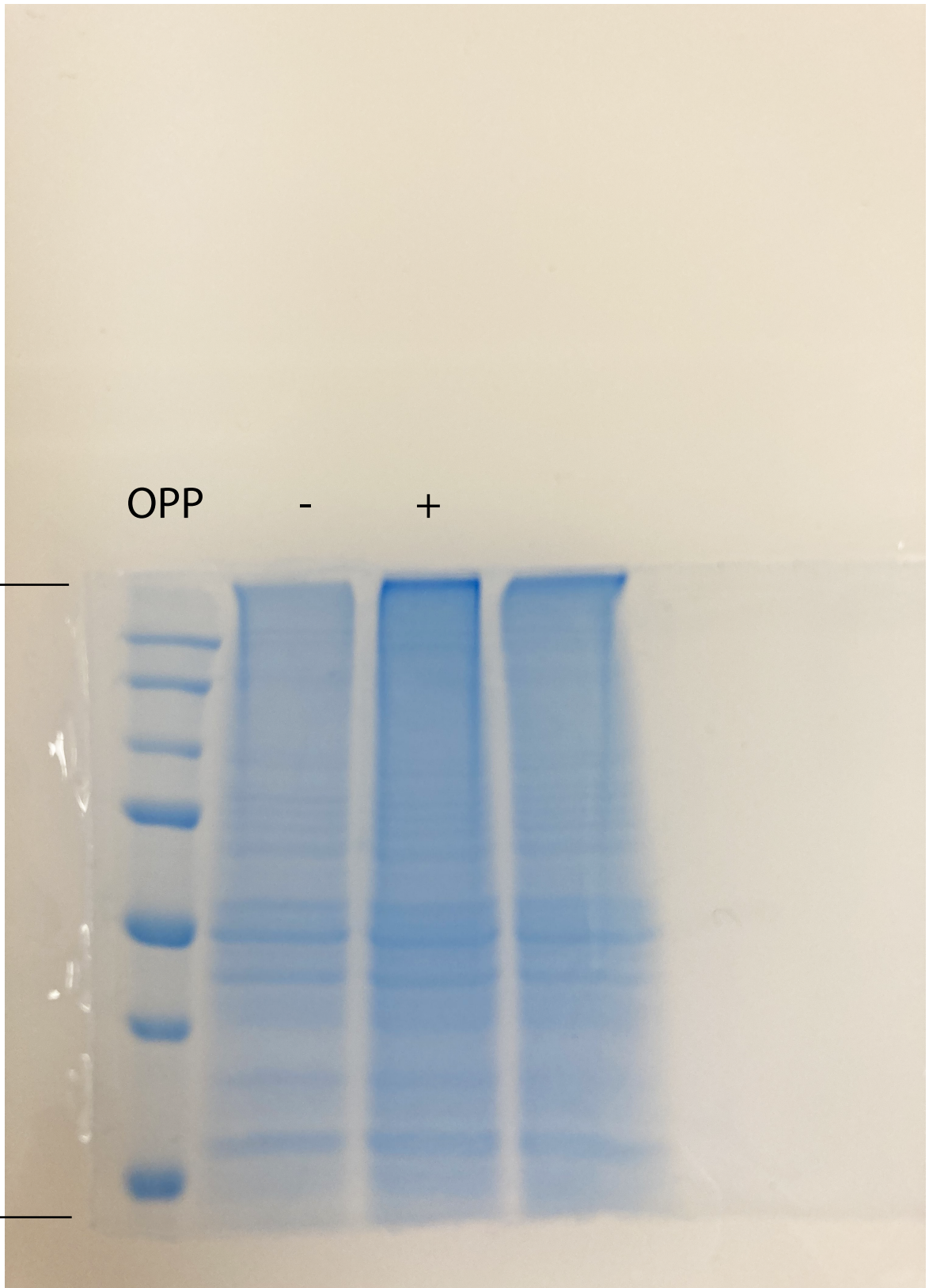

Supplement: Figure 1—source data 1. [file elife-83545-fig1-data1.zip › Figure 1/Fig_1E_total_prot_annotated.pdf]

OPP

-

+

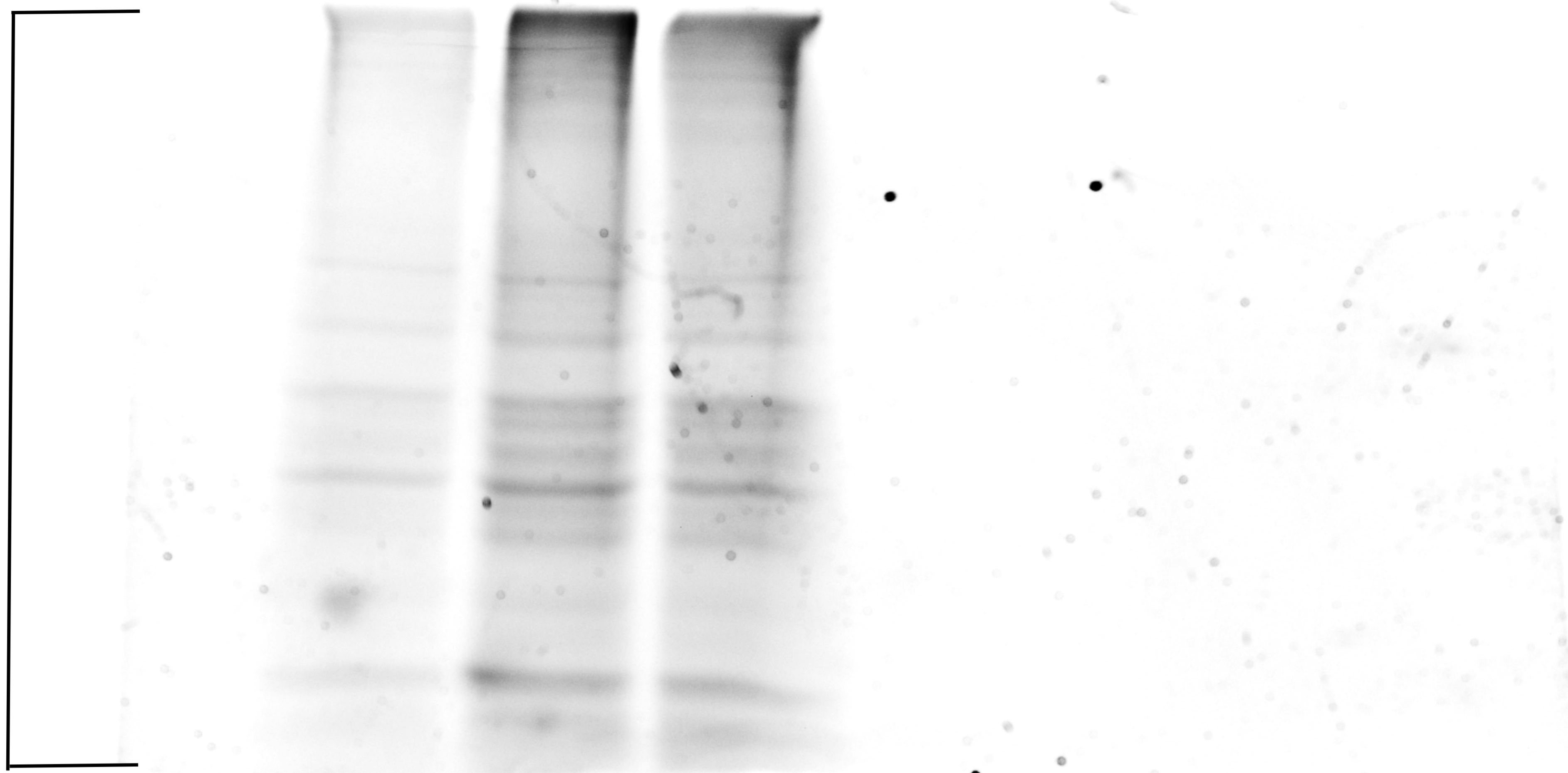

Supplement: Figure 1—source data 1. [file elife-83545-fig1-data1.zip › Figure 1/Fig_1E_fluorescence_annotated.pdf]

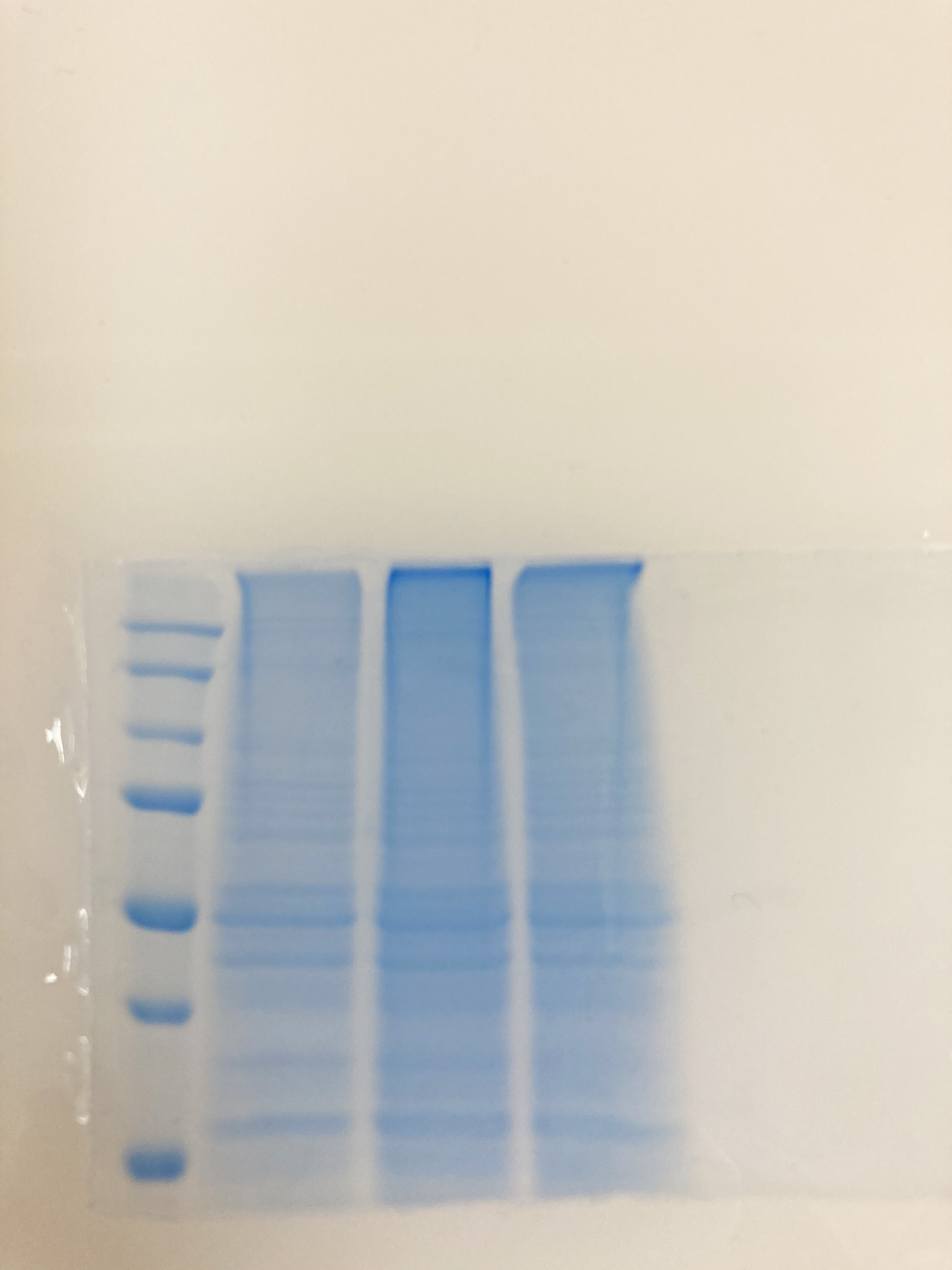

Supplement: Figure 1—source data 1. [file elife-83545-fig1-data1.zip › Figure 1/Fig_1E_total_prot_original.jpeg]

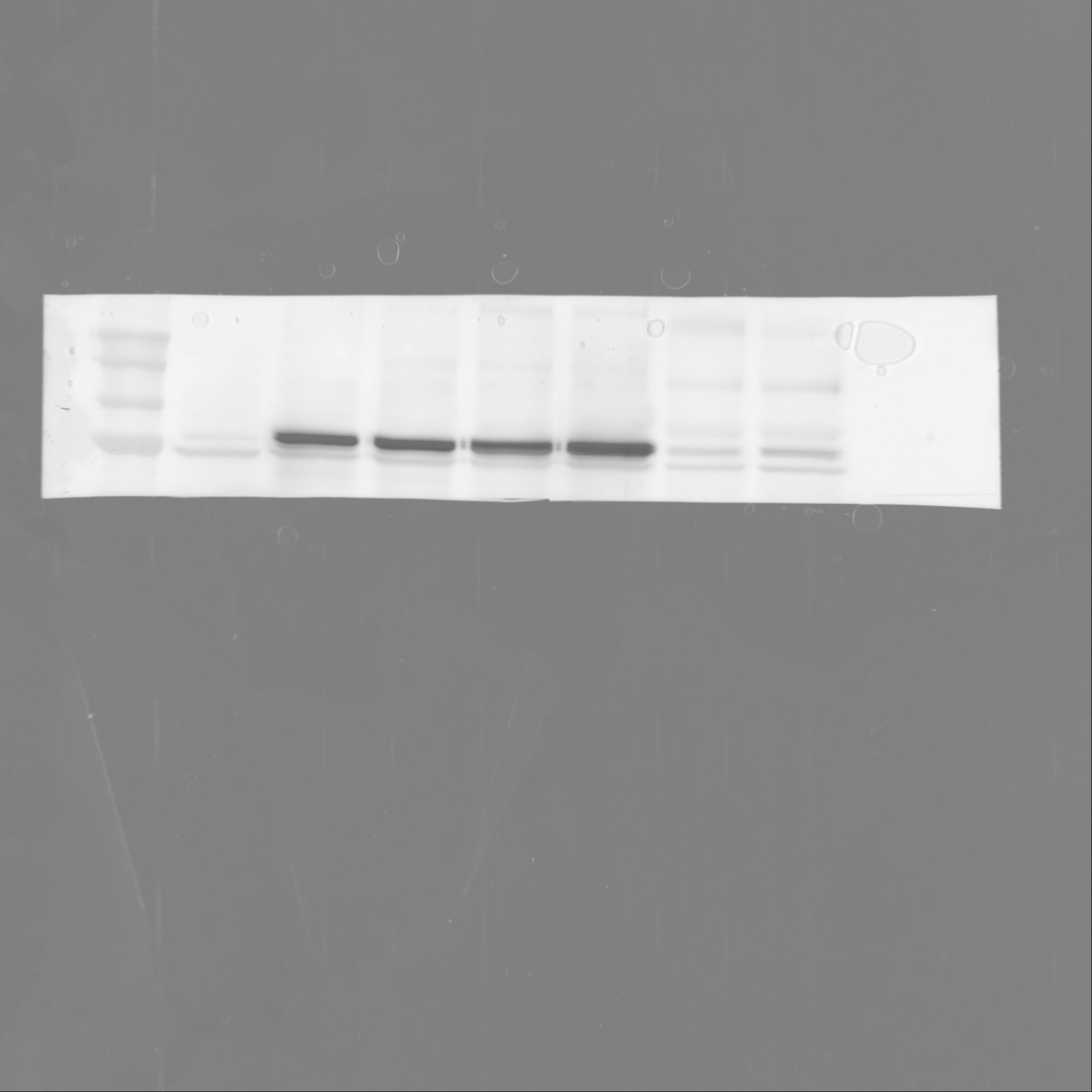

Supplement: Figure 1—figure supplement 1—source data 1. [file elife-83545-fig1-figsupp1-data1.zip › Figure 1-fig supp 1/Fig_1-S1C_FLAG_original.tiff]

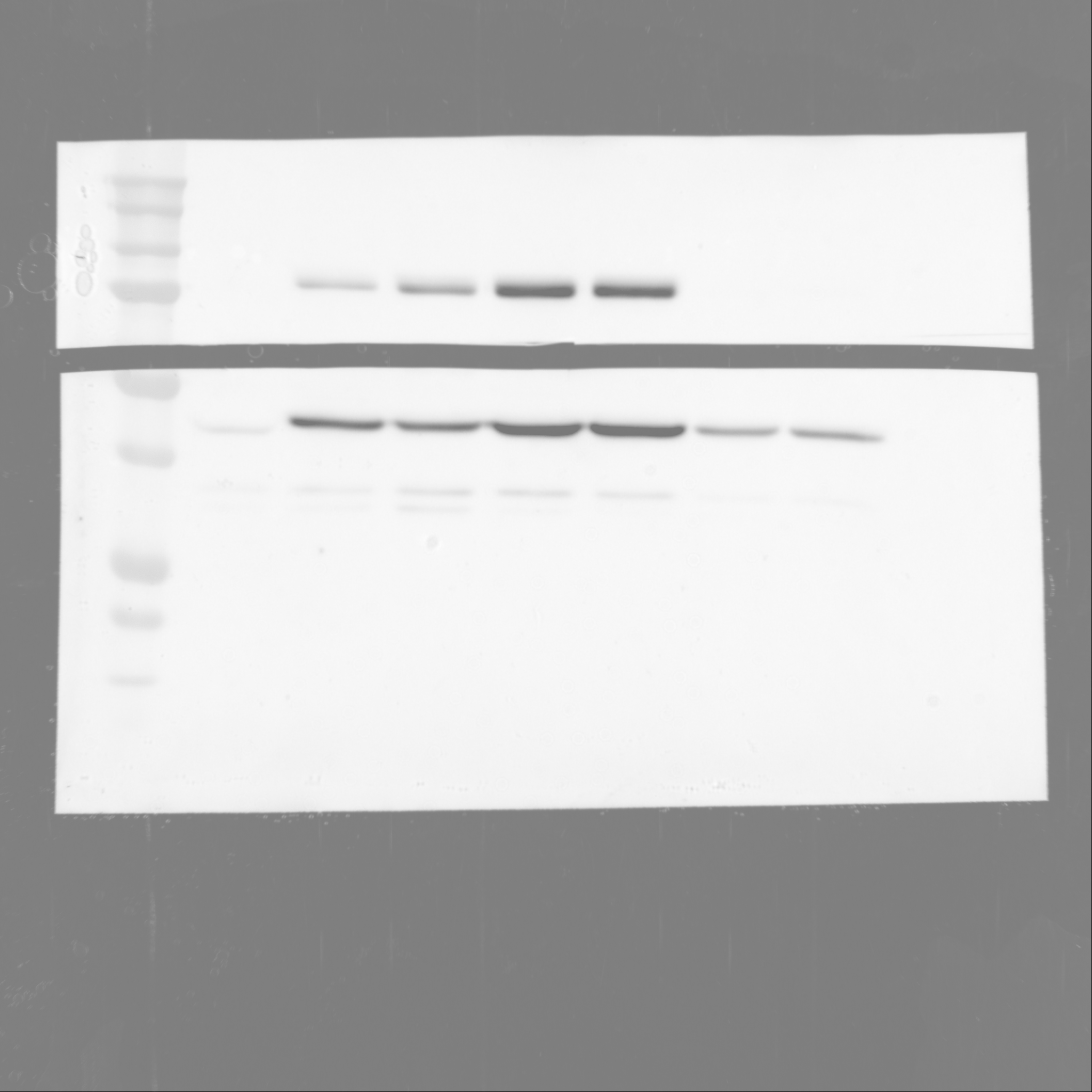

Supplement: Figure 1—figure supplement 1—source data 1. [file elife-83545-fig1-figsupp1-data1.zip › Figure 1-fig supp 1/Fig_1-S1C_actin_original.tiff]

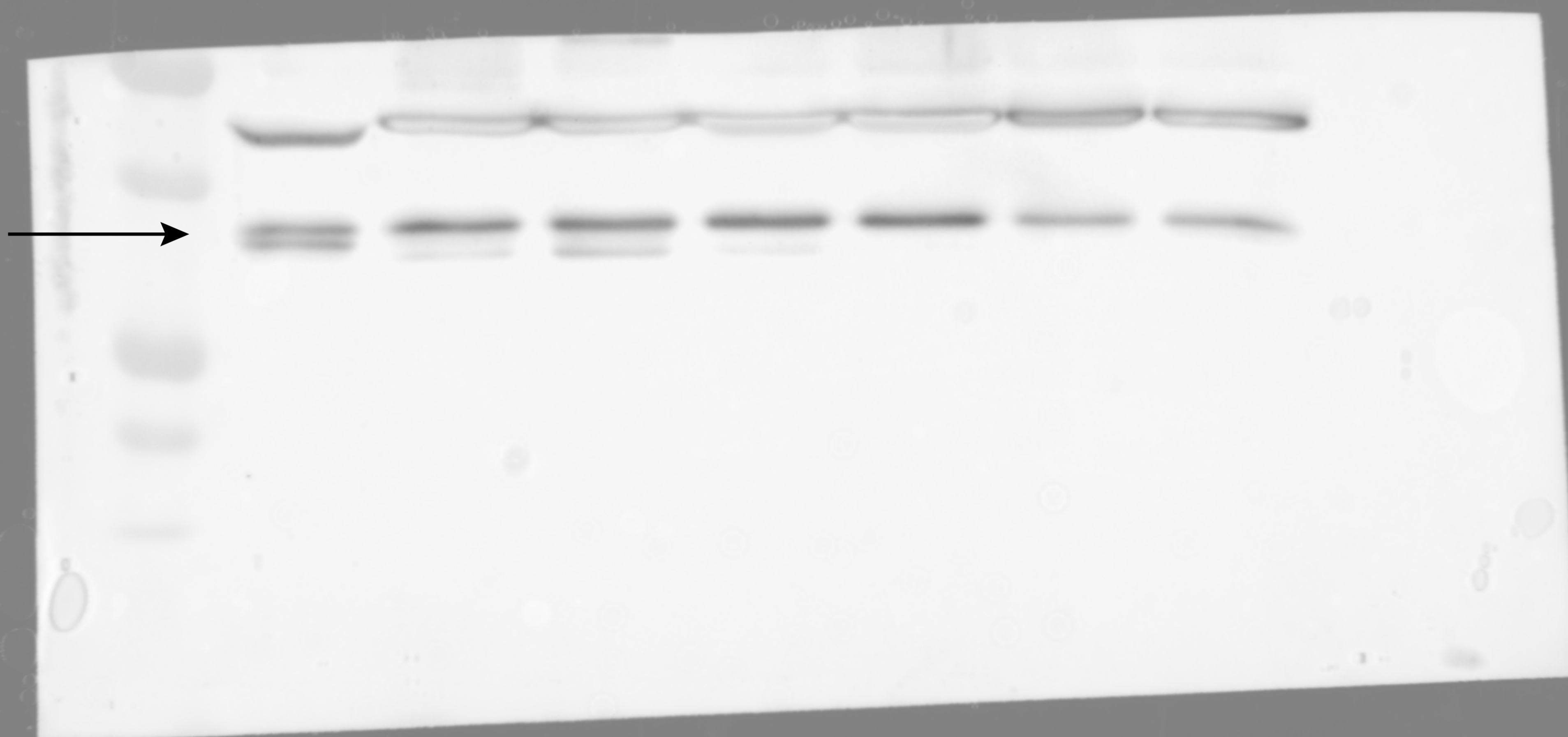

Supplement: Figure 1—figure supplement 1—source data 1. [file elife-83545-fig1-figsupp1-data1.zip › Figure 1-fig supp 1/Fig_1-S1C_GAPDH_annotated.pdf]

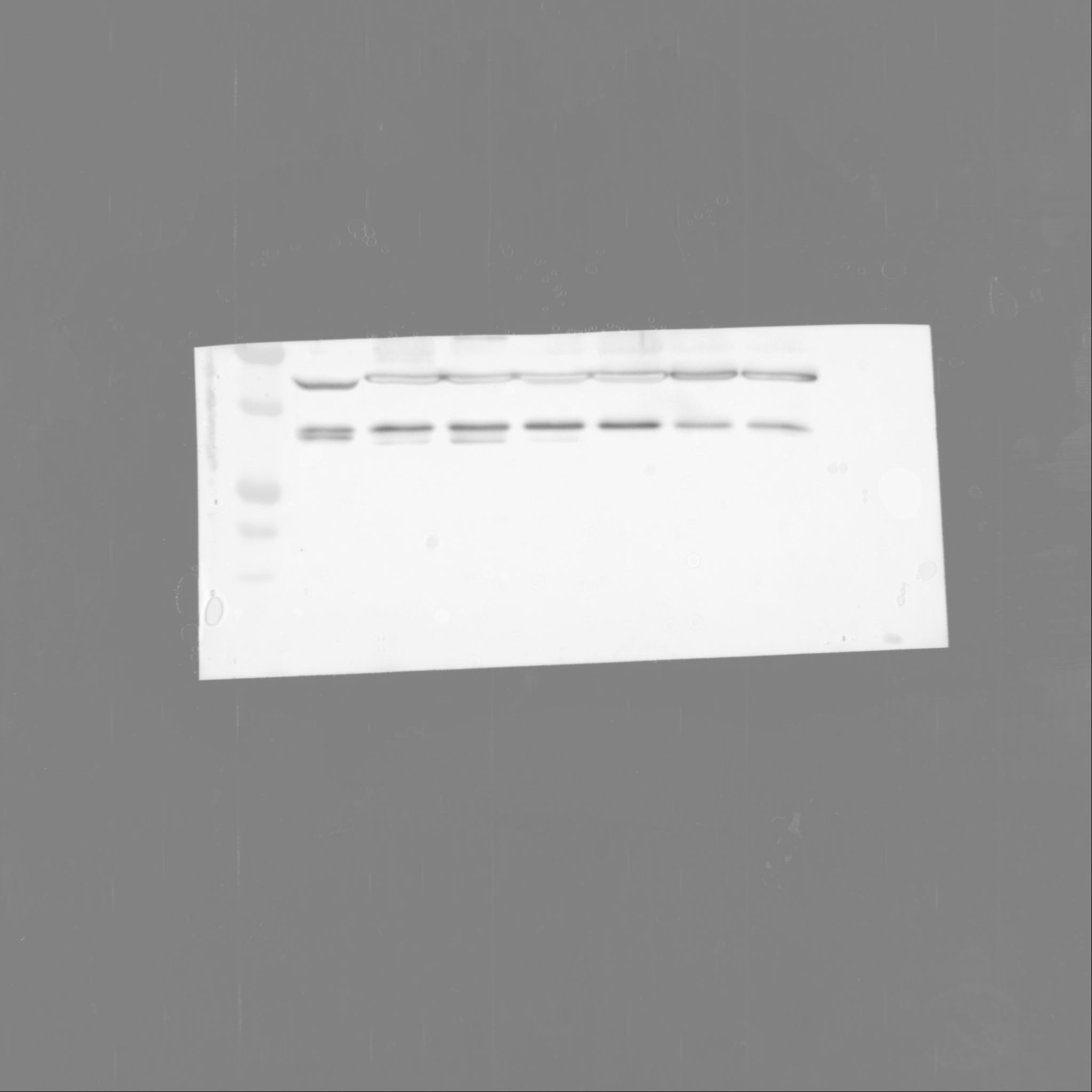

Supplement: Figure 1—figure supplement 1—source data 1. [file elife-83545-fig1-figsupp1-data1.zip › Figure 1-fig supp 1/Fig_1-S1C_GAPDH_original.tiff]

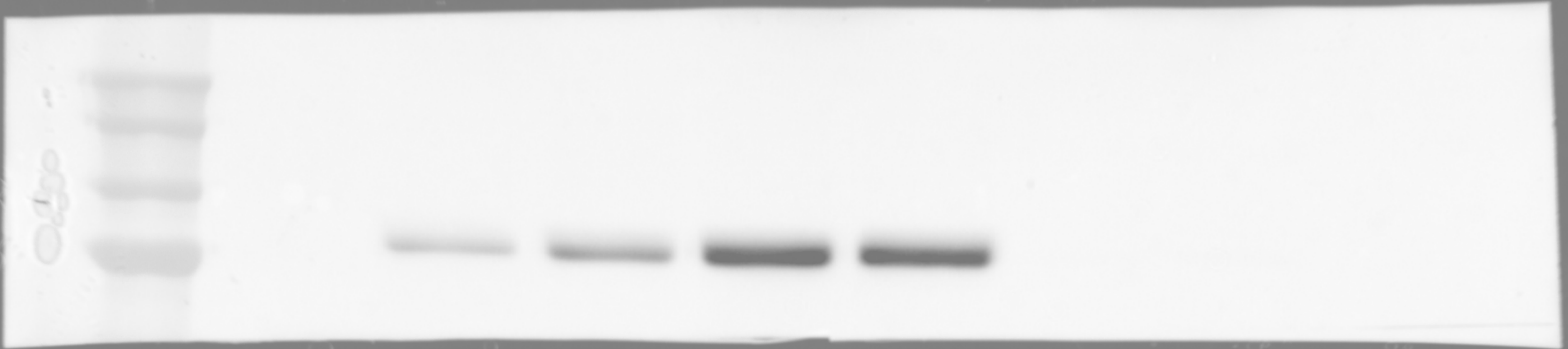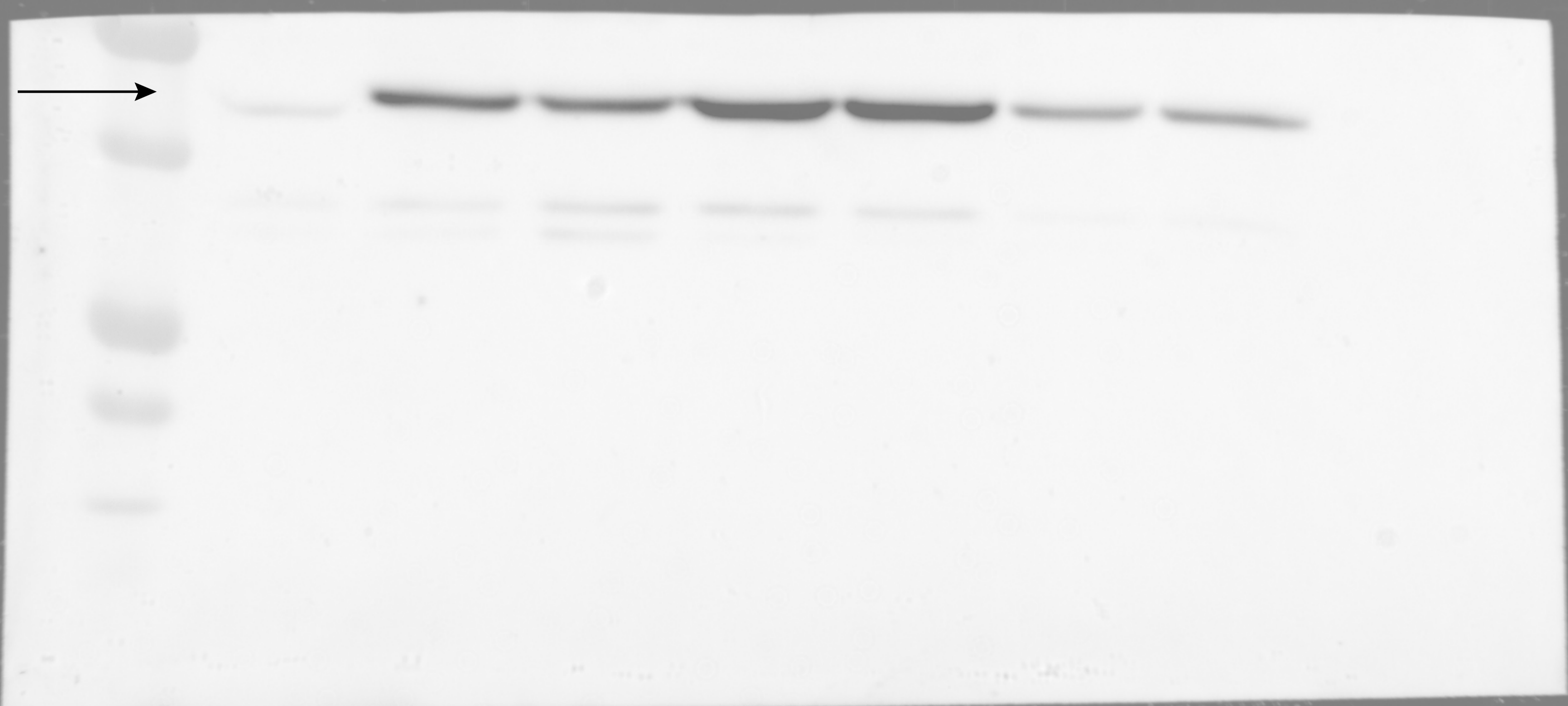

Supplement: Figure 1—figure supplement 1—source data 1. [file elife-83545-fig1-figsupp1-data1.zip › Figure 1-fig supp 1/Fig_1-S1C_actin_annotated.pdf]

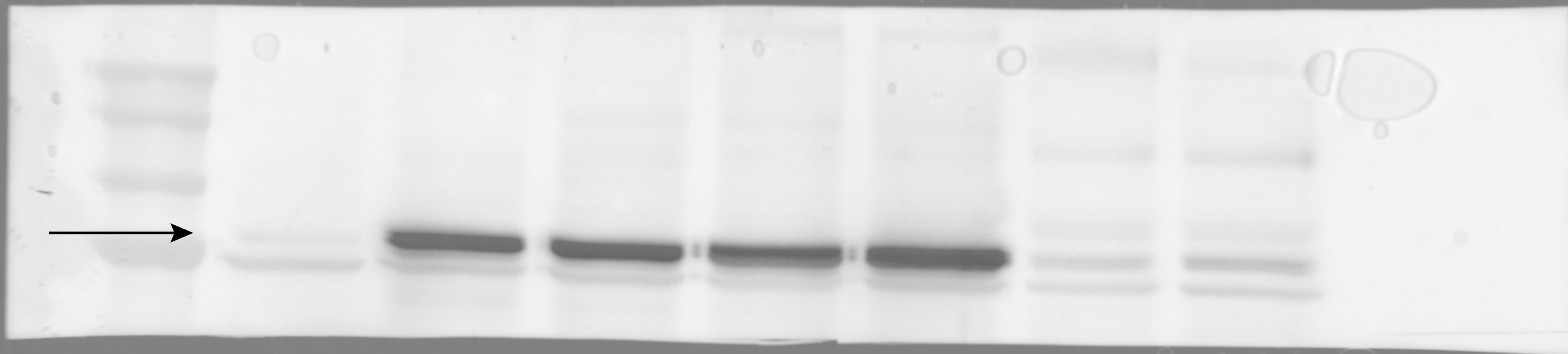

Supplement: Figure 1—figure supplement 1—source data 1. [file elife-83545-fig1-figsupp1-data1.zip › Figure 1-fig supp 1/Fig_1-S1C_FLAG_annotated.pdf]

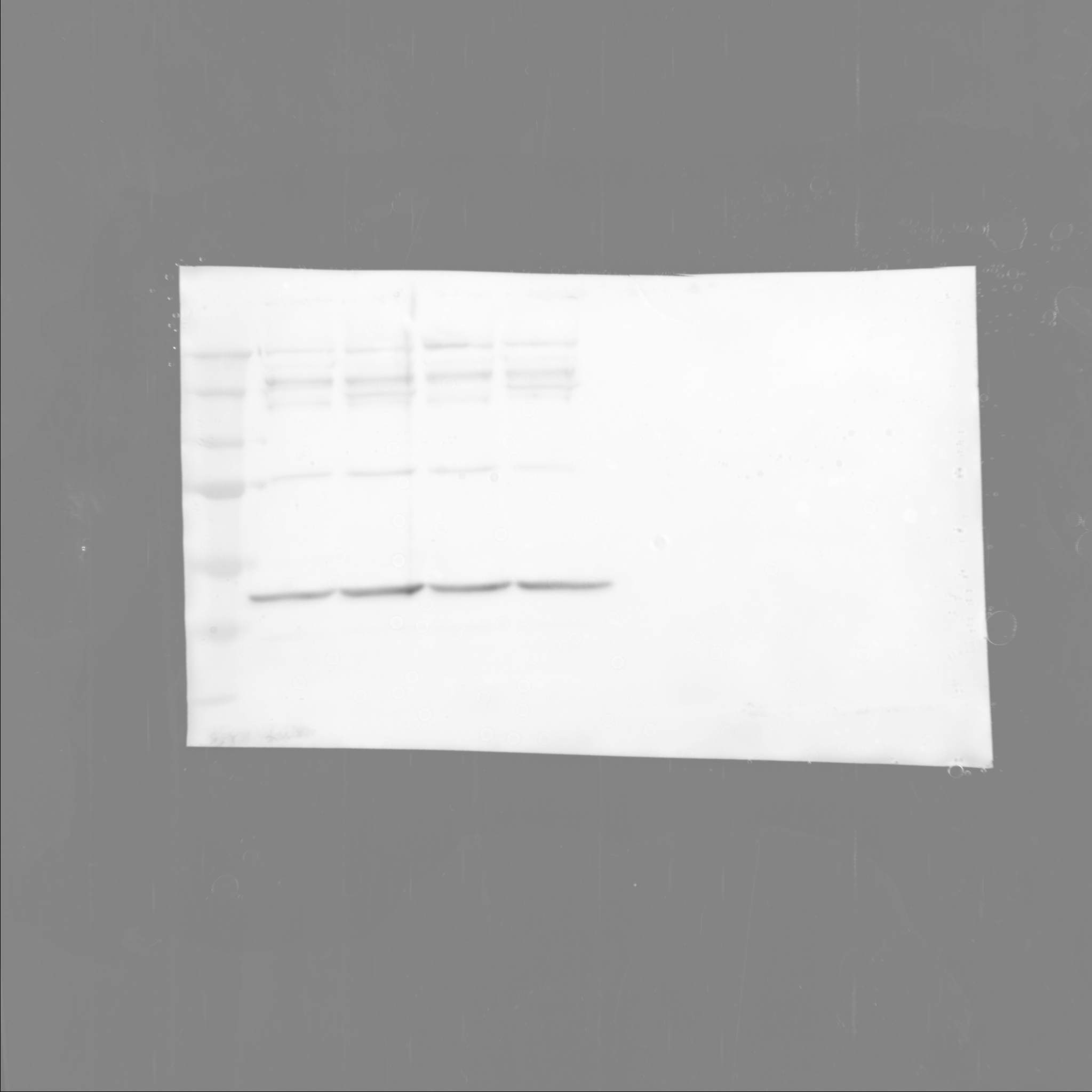

Supplement: Figure 2—source data 1. [file elife-83545-fig2-data1.zip › Figure 2/Fig_2F_actin_original.tif]

PhAc-OPP

0

50

100

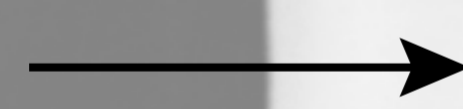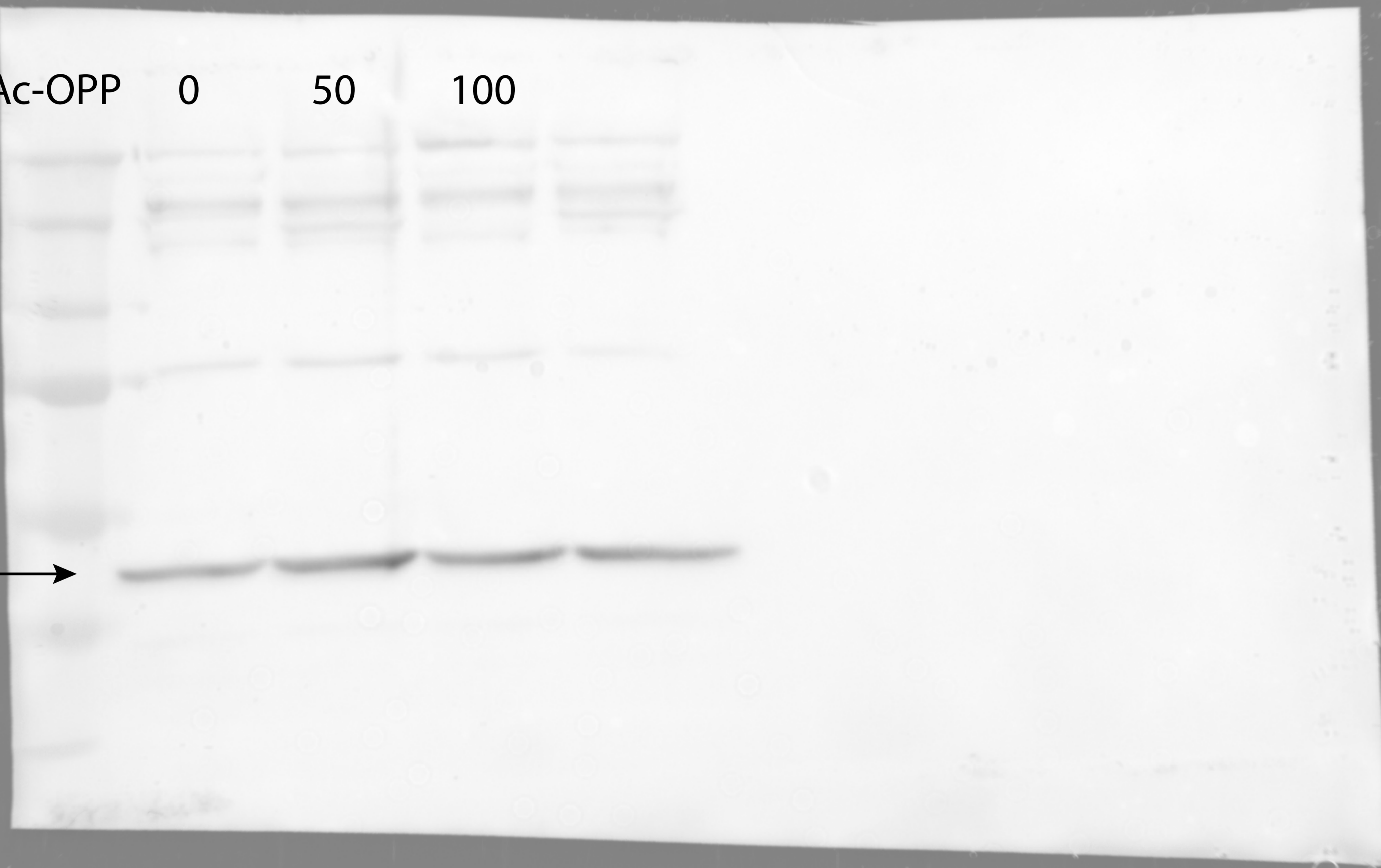

Supplement: Figure 2—source data 1. [file elife-83545-fig2-data1.zip › Figure 2/Fig_2F_actin_annotated.pdf]

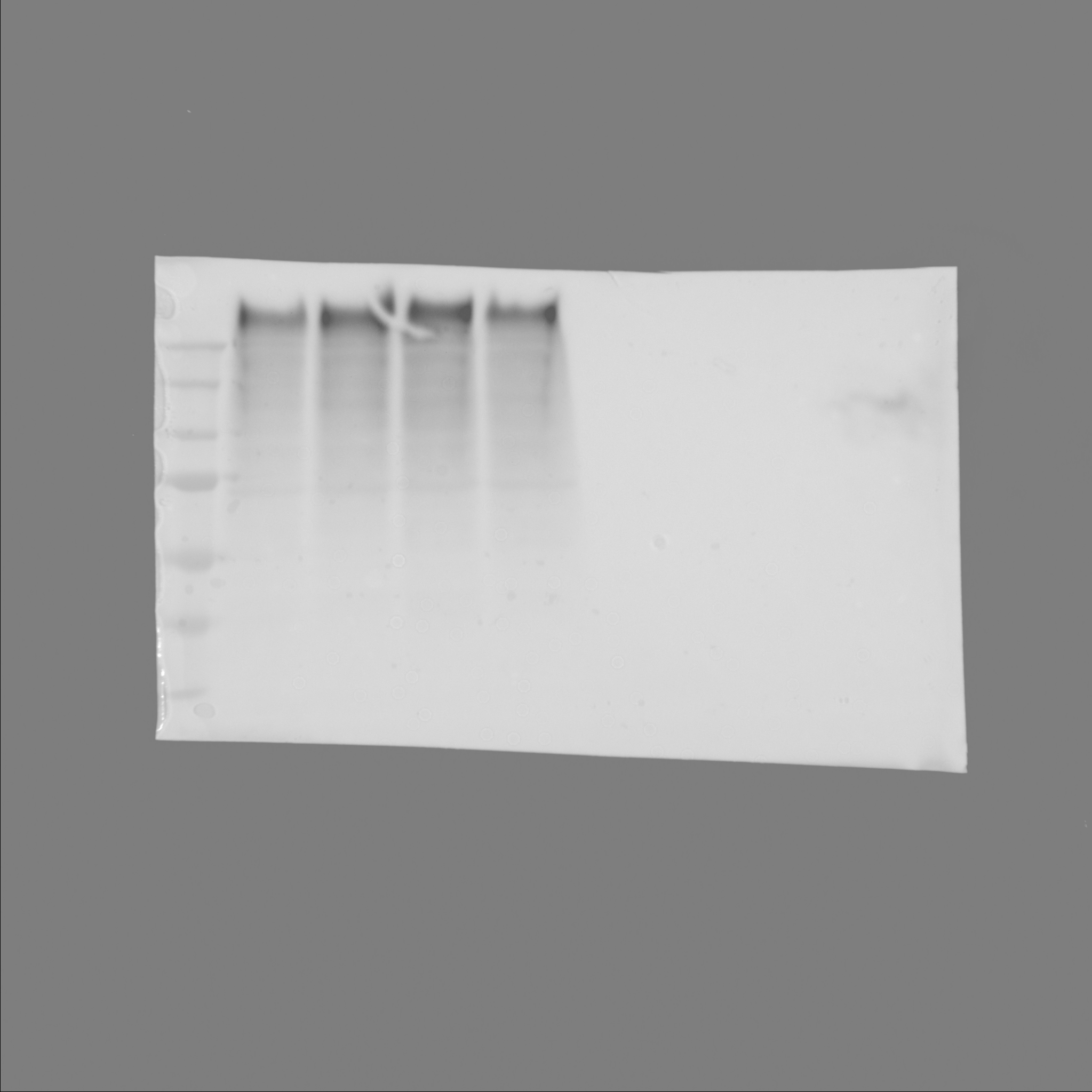

Supplement: Figure 2—source data 1. [file elife-83545-fig2-data1.zip › Figure 2/Fig_2F_ubiquitin_original.tif]

PhAc-OPP

0

50

100

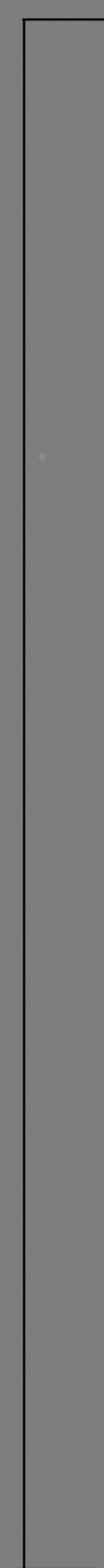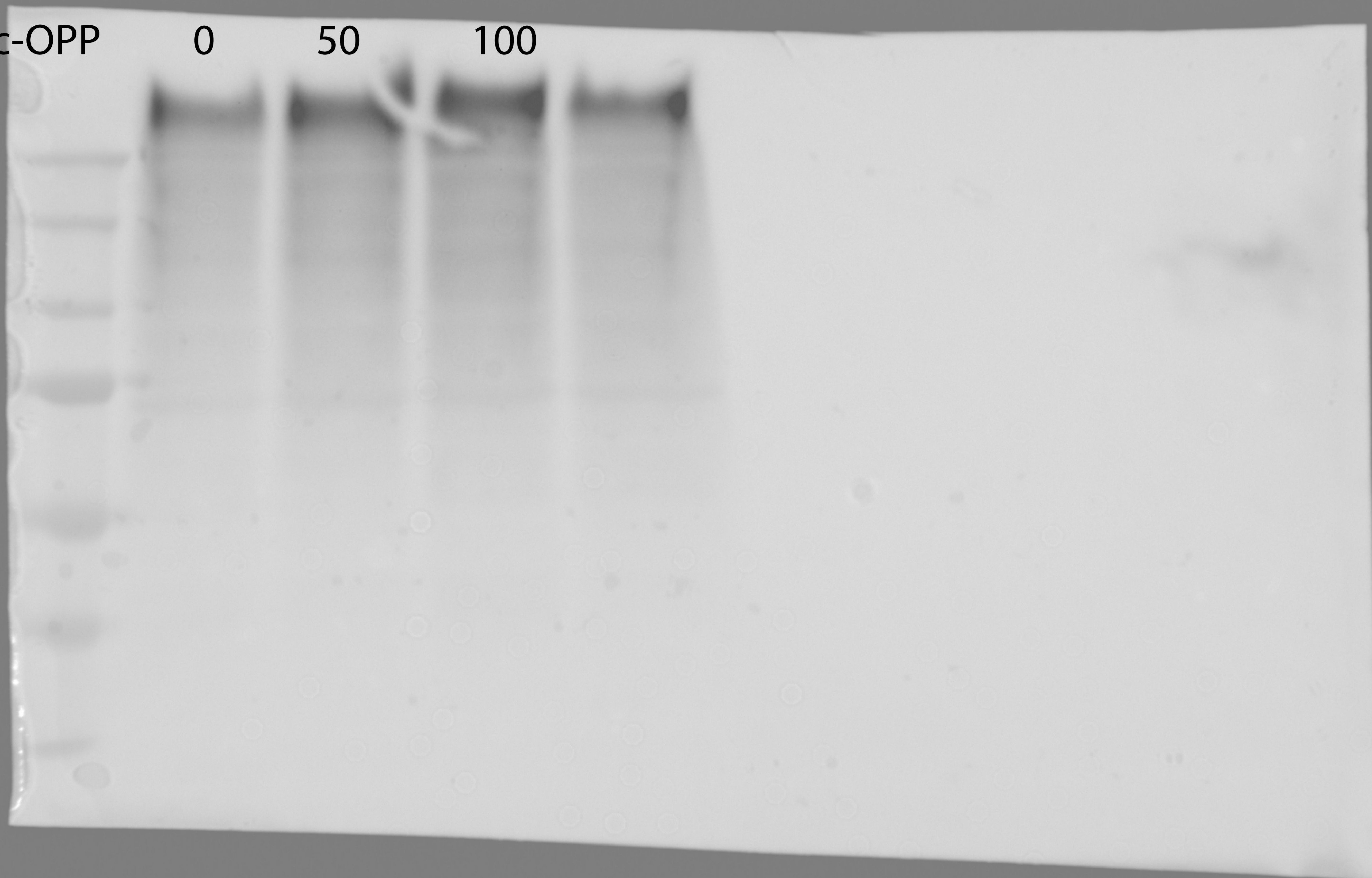

Supplement: Figure 2—source data 1. [file elife-83545-fig2-data1.zip › Figure 2/Fig_2F_ubiquitin_annotated.pdf]

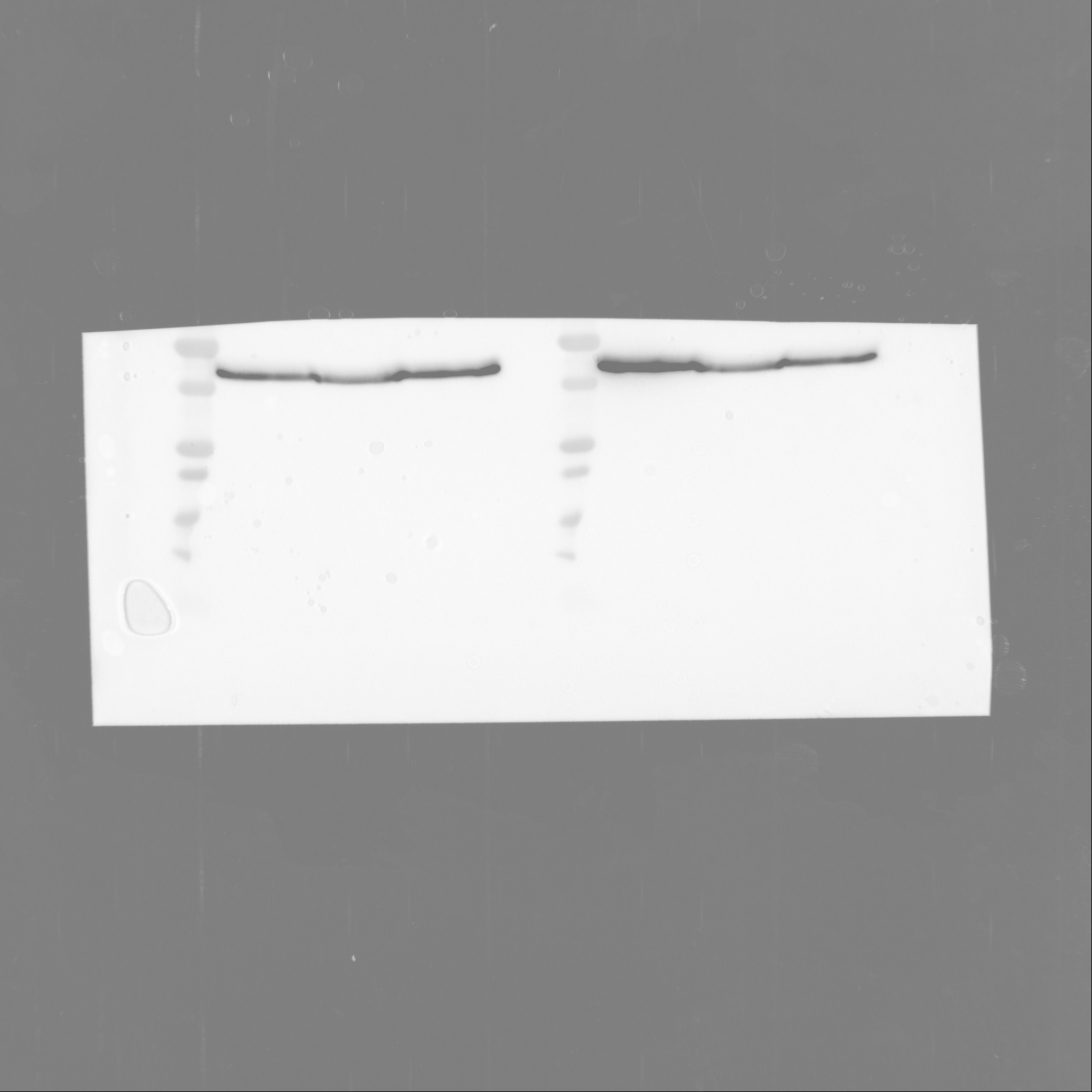

Supplement: Figure 2—figure supplement 1—source data 1. [file elife-83545-fig2-figsupp1-data1.zip › Figure 2-fig supp 1/Fig_2-S1A_actin_original.tiff]

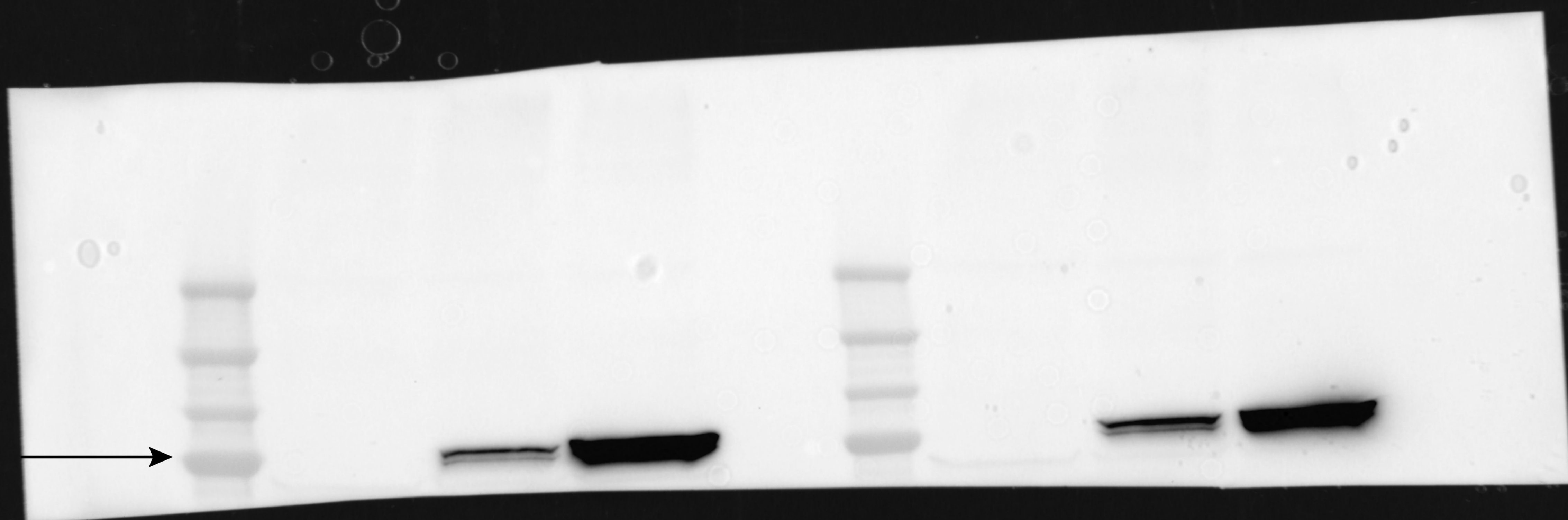

Supplement: Figure 2—figure supplement 1—source data 1. [file elife-83545-fig2-figsupp1-data1.zip › Figure 2-fig supp 1/Fig_2-S1A_FLAG_annotated.pdf]

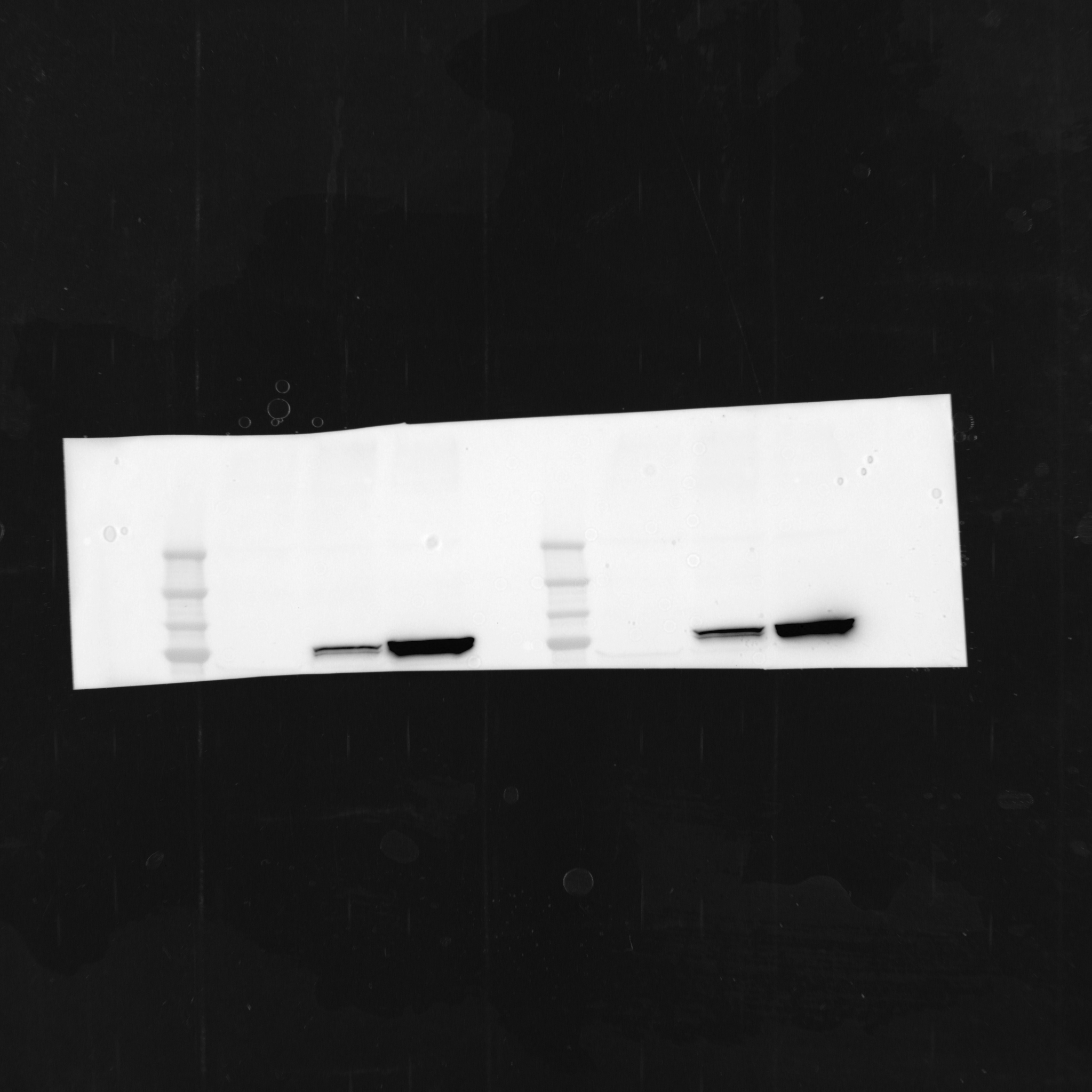

Supplement: Figure 2—figure supplement 1—source data 1. [file elife-83545-fig2-figsupp1-data1.zip › Figure 2-fig supp 1/Fig_2-S1A_FLAG_original.tiff]

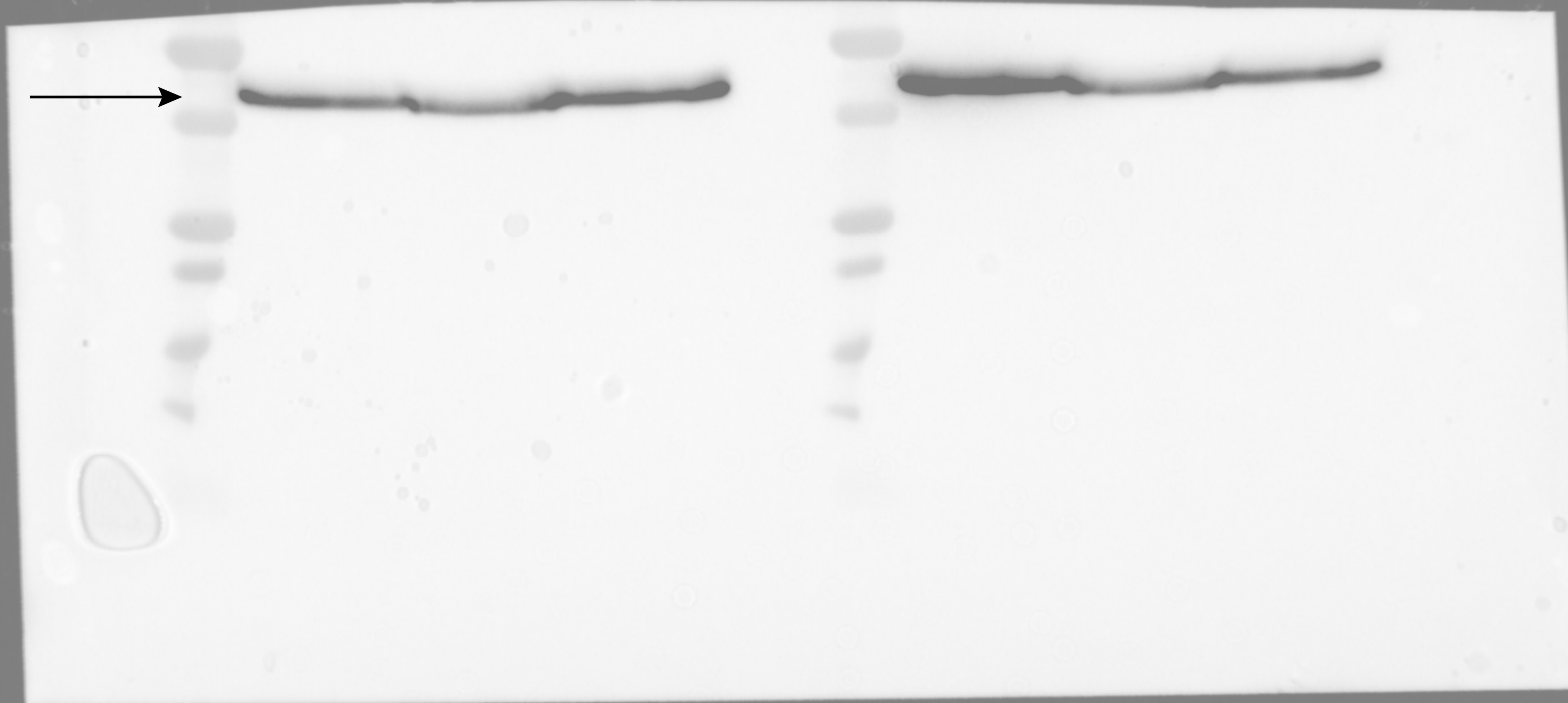

Supplement: Figure 2—figure supplement 1—source data 1. [file elife-83545-fig2-figsupp1-data1.zip › Figure 2-fig supp 1/Fig_2-S1A_actin_annotated.pdf]

INPUT                      ELUATE

PhAc-OPP    -            +            -            +

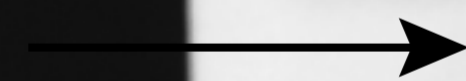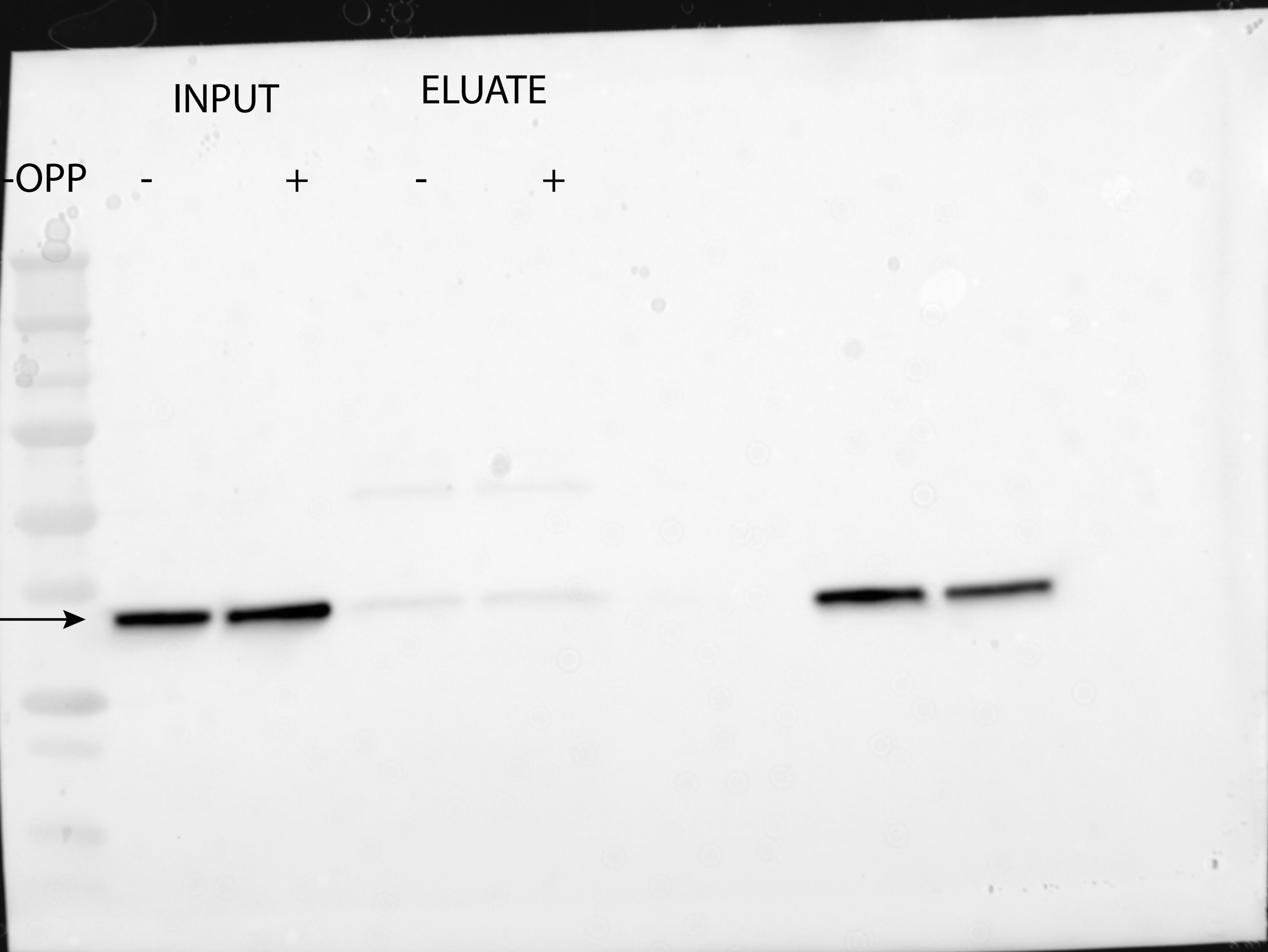

Supplement: Figure 4—source data 1. [file elife-83545-fig4-data1.zip › Figure 4/Fig_4F_syntaxin_annotated.pdf]

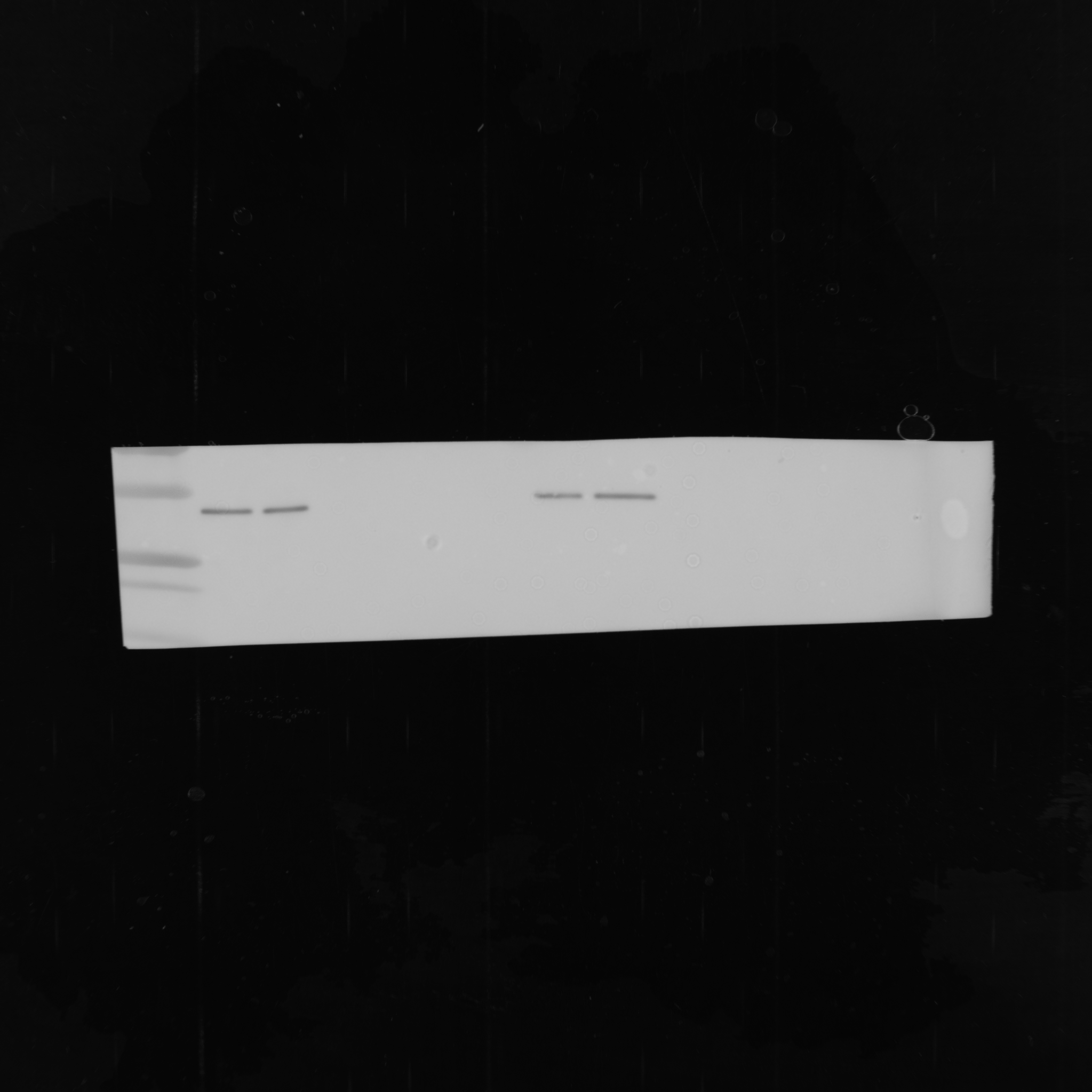

Supplement: Figure 4—source data 1. [file elife-83545-fig4-data1.zip › Figure 4/Fig_4C_syntaxin_input_original.tiff]

PhAc-OPP

ELUATE

-

+

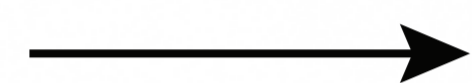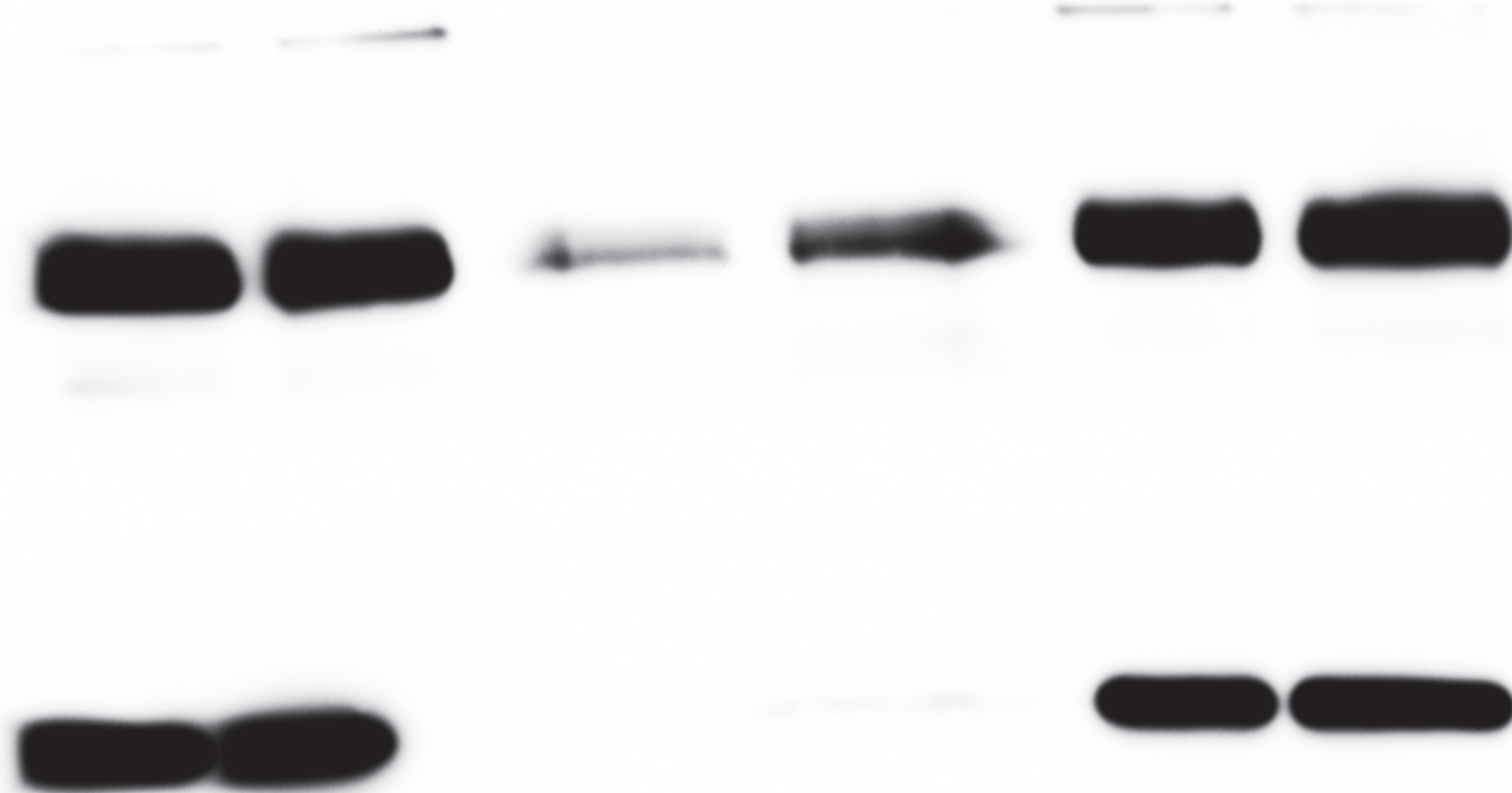

Supplement: Figure 4—source data 1. [file elife-83545-fig4-data1.zip › Figure 4/Fig_4C_synapsin_annotated.pdf]

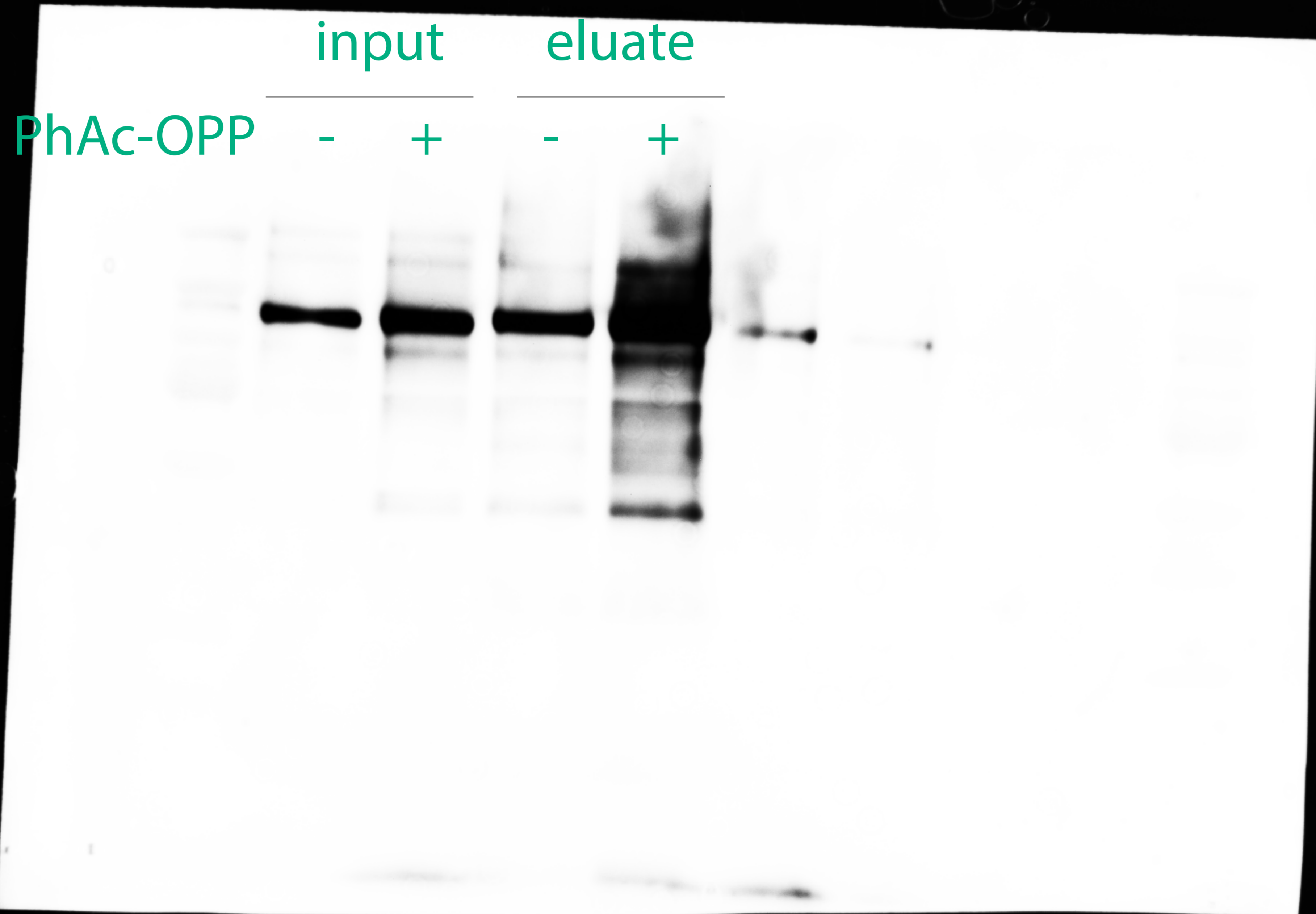

Supplement: Figure 4—source data 1. [file elife-83545-fig4-data1.zip › Figure 4/Fig_4E_biotin_annotated.pdf]

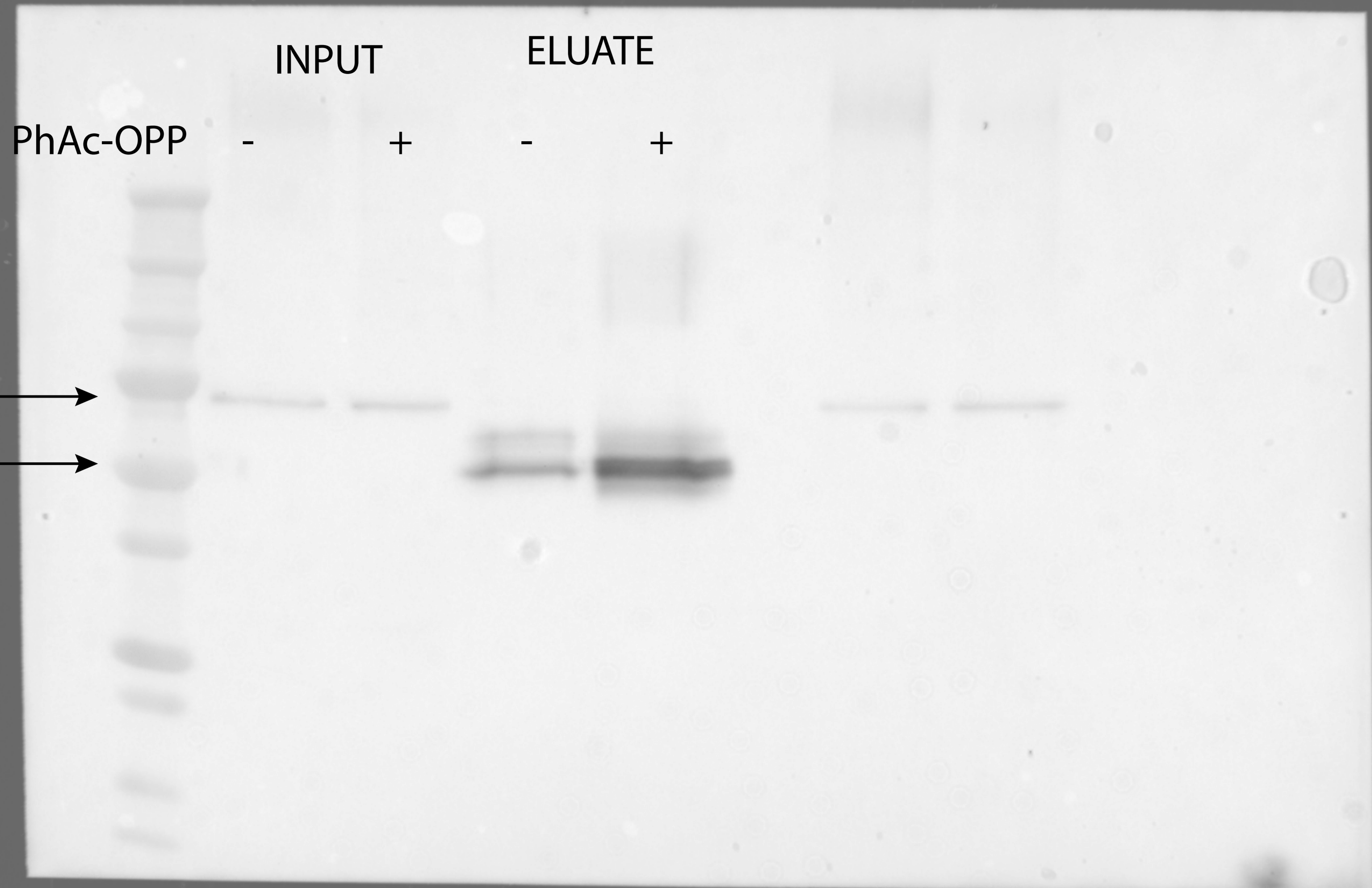

Supplement: Figure 4—source data 1. [file elife-83545-fig4-data1.zip › Figure 4/Fig_4F_draper_annotated.pdf]

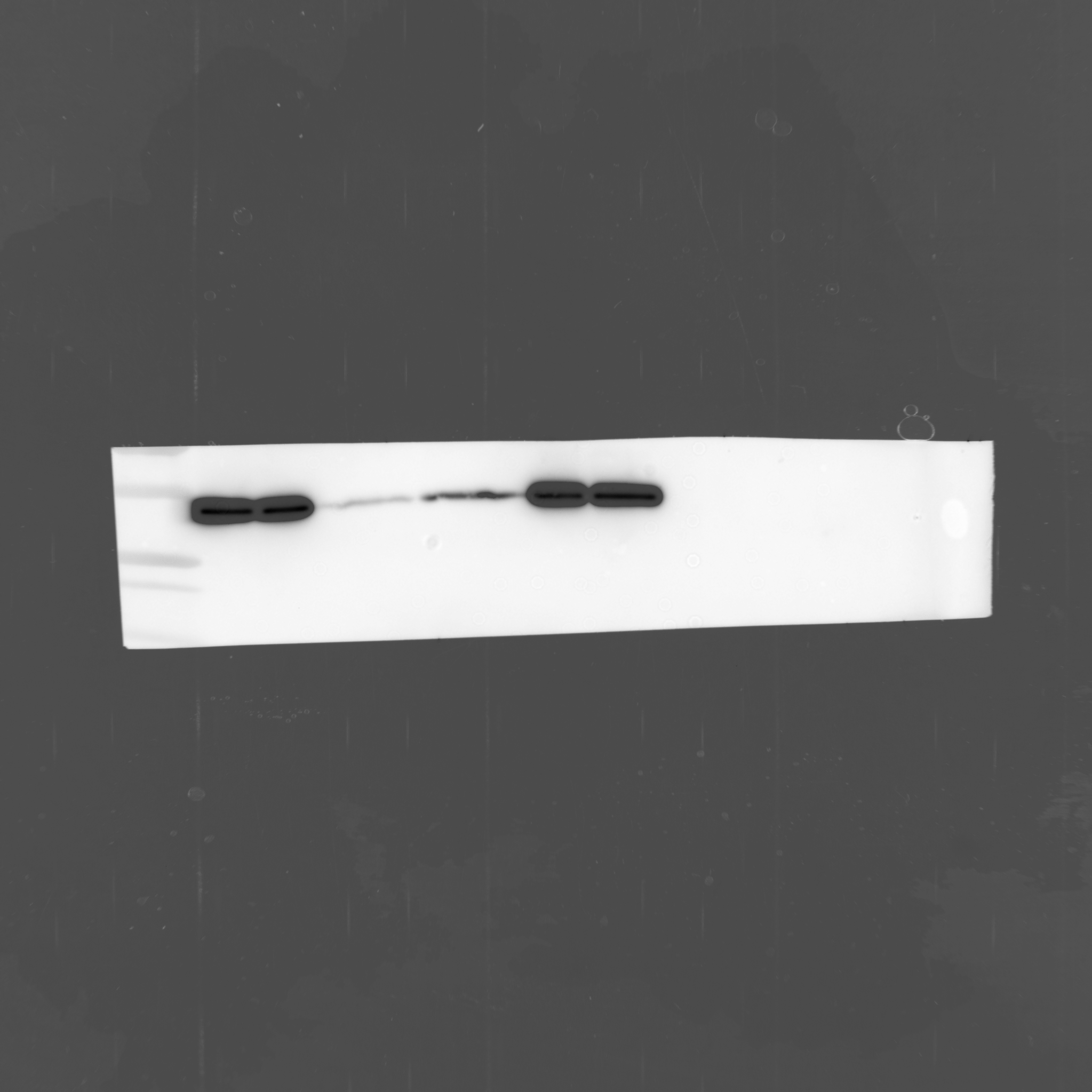

Supplement: Figure 4—source data 1. [file elife-83545-fig4-data1.zip › Figure 4/Fig_4C_syntaxin_eluate_original.tiff]

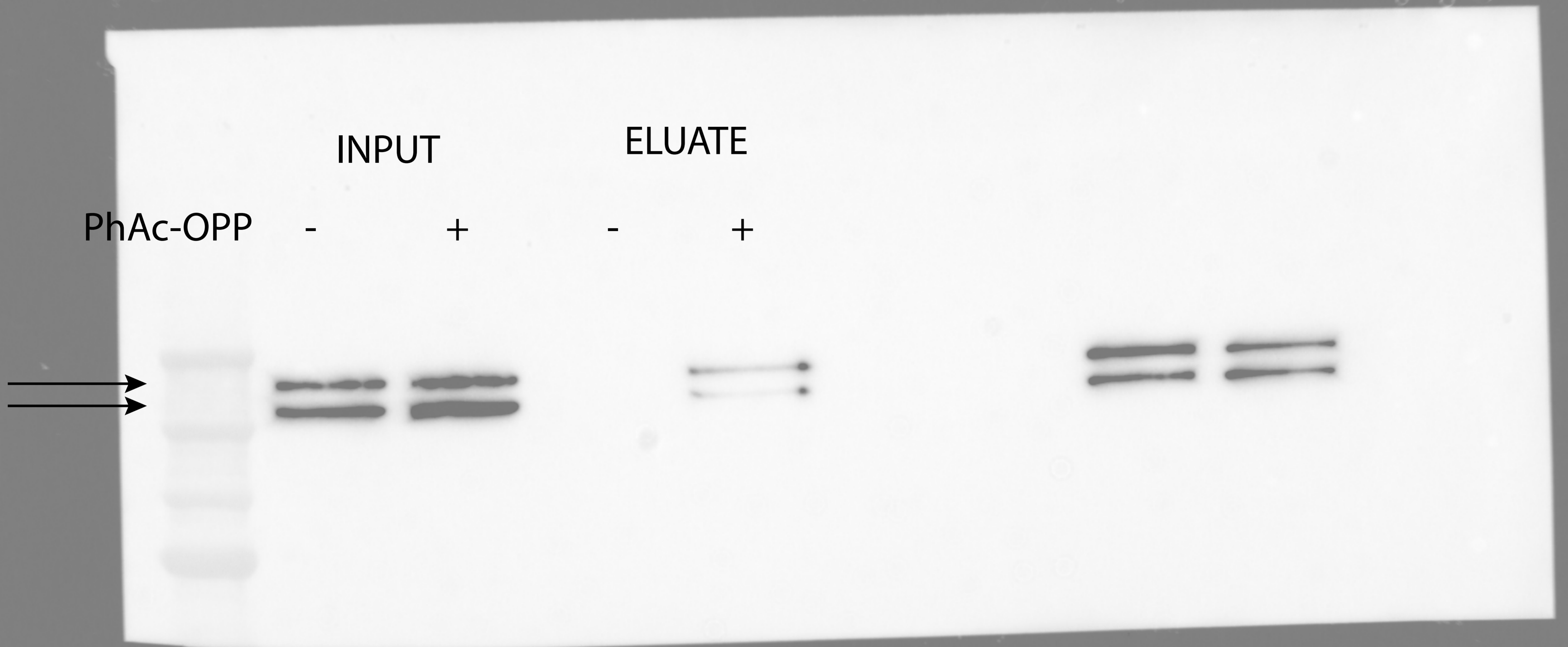

Supplement: Figure 4—source data 1. [file elife-83545-fig4-data1.zip › Figure 4/Fig_4C_brp_annotated.pdf]

PhAc-OPP

|  | input |   | eluate |   |
|--|-------|---|--------|---|
|  | -     | + | -      | + |

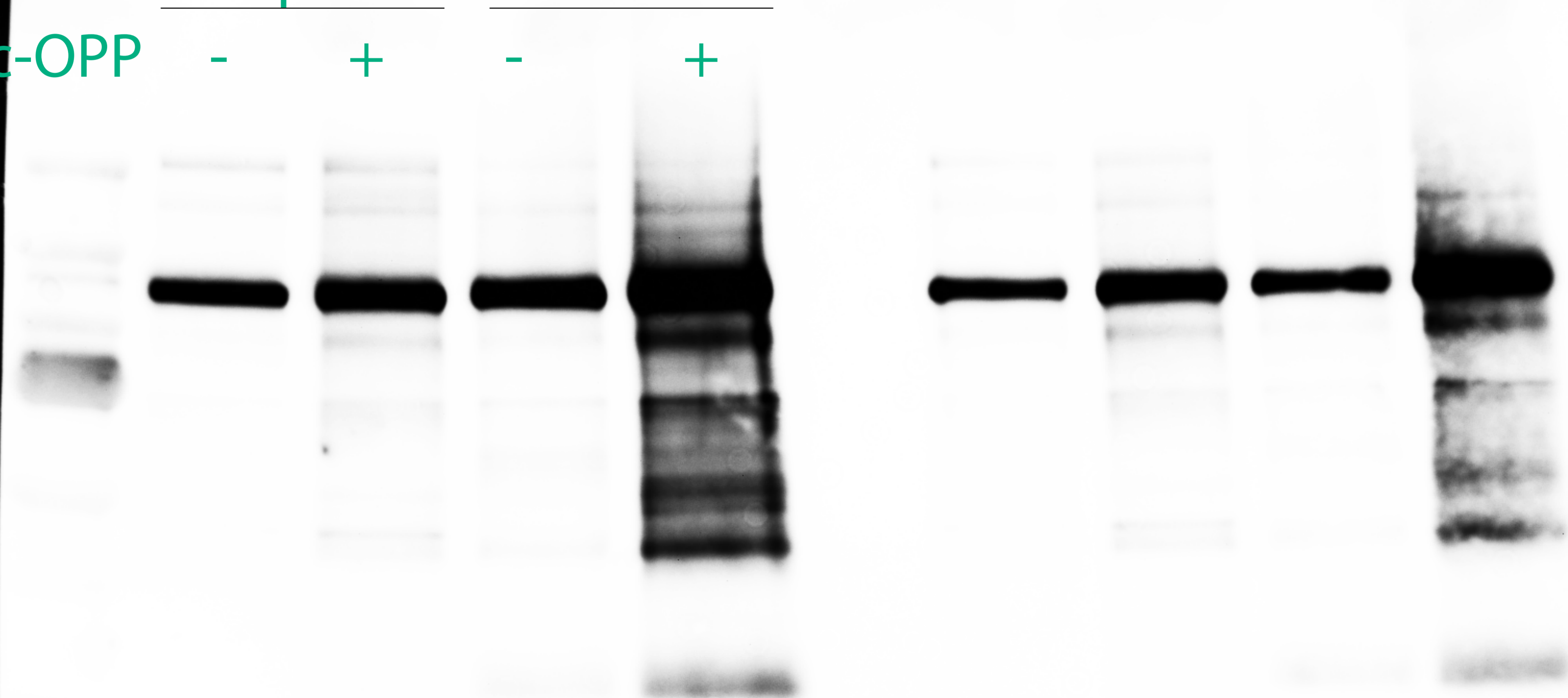

Supplement: Figure 4—source data 1. [file elife-83545-fig4-data1.zip › Figure 4/Fig_4B_biotin_annotated.pdf]

ELUATE

PhAc-OPP

-

+

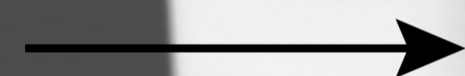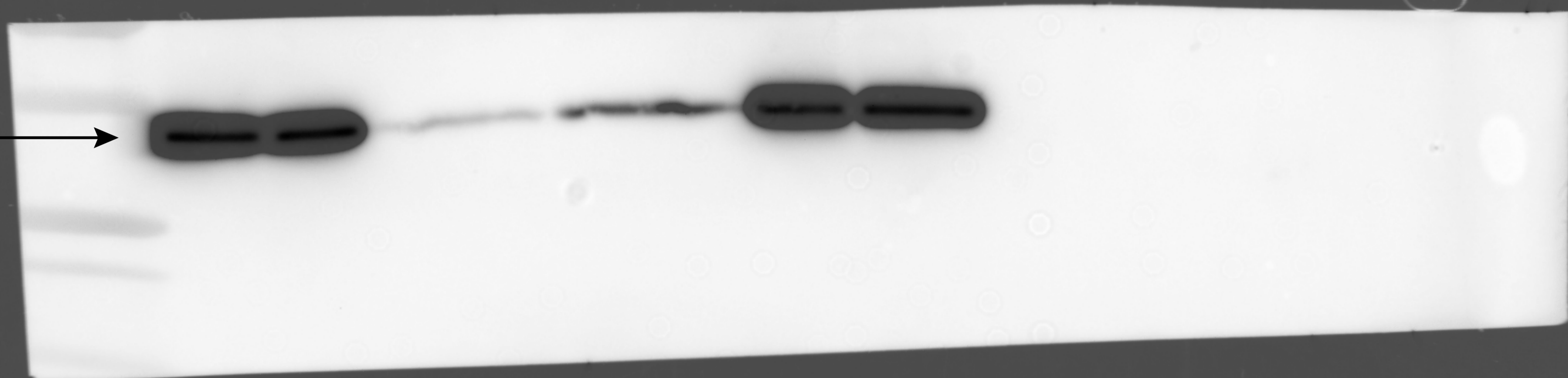

Supplement: Figure 4—source data 1. [file elife-83545-fig4-data1.zip › Figure 4/Fig_4C_syntaxin_eluate_annotated.pdf]

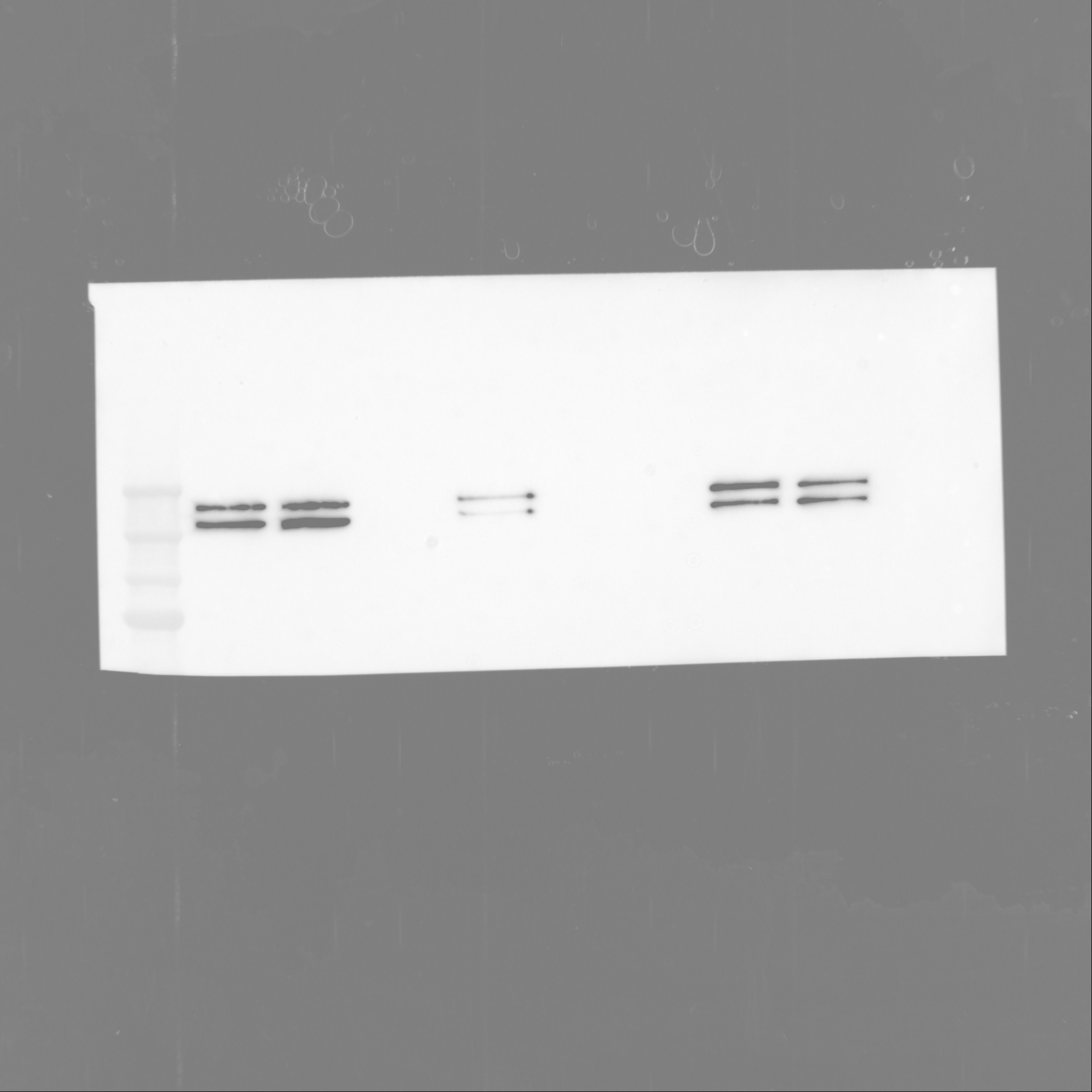

Supplement: Figure 4—source data 1. [file elife-83545-fig4-data1.zip › Figure 4/Fig_4C_brp_original.tiff]

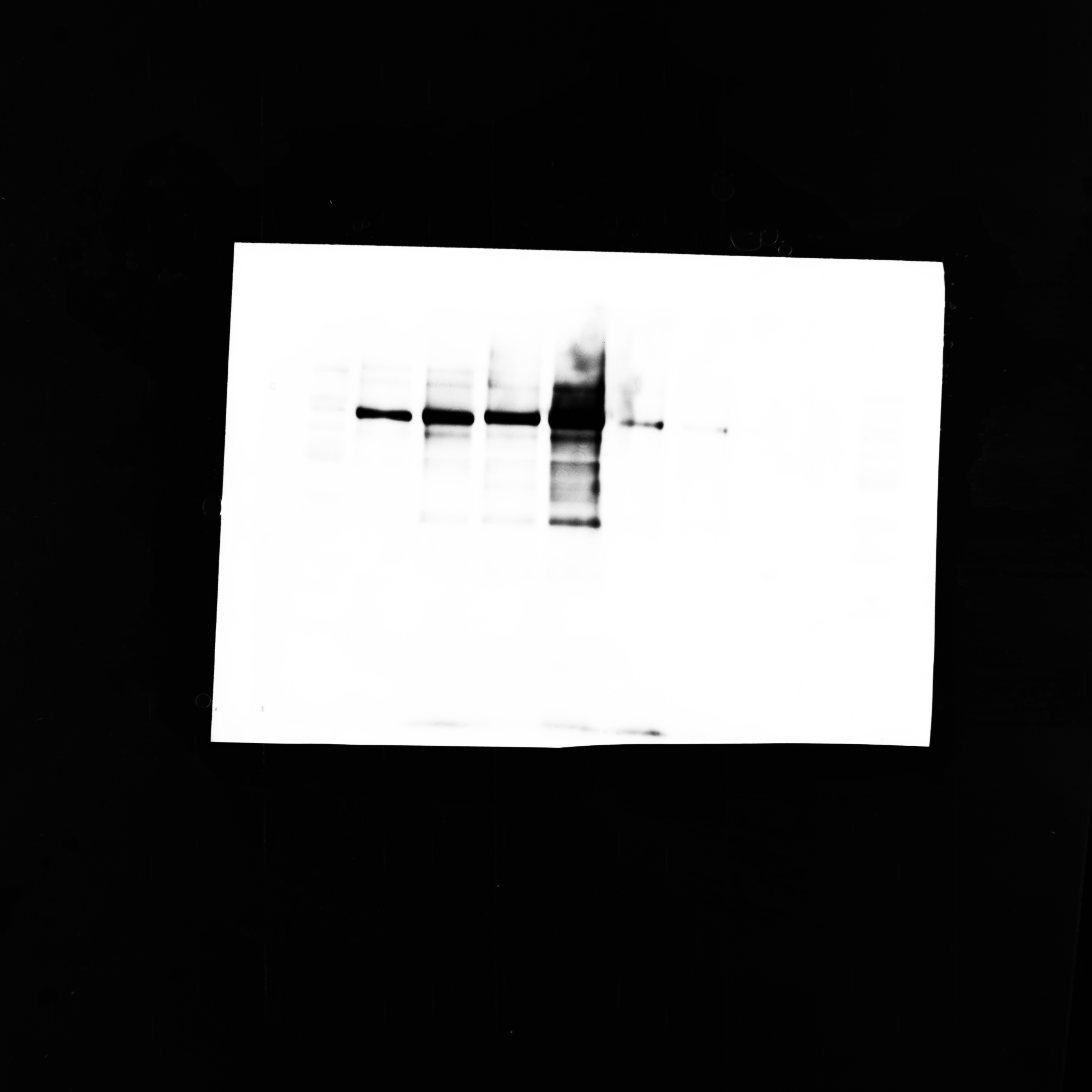

Supplement: Figure 4—source data 1. [file elife-83545-fig4-data1.zip › Figure 4/Fig_4E_biotin_original.tif]

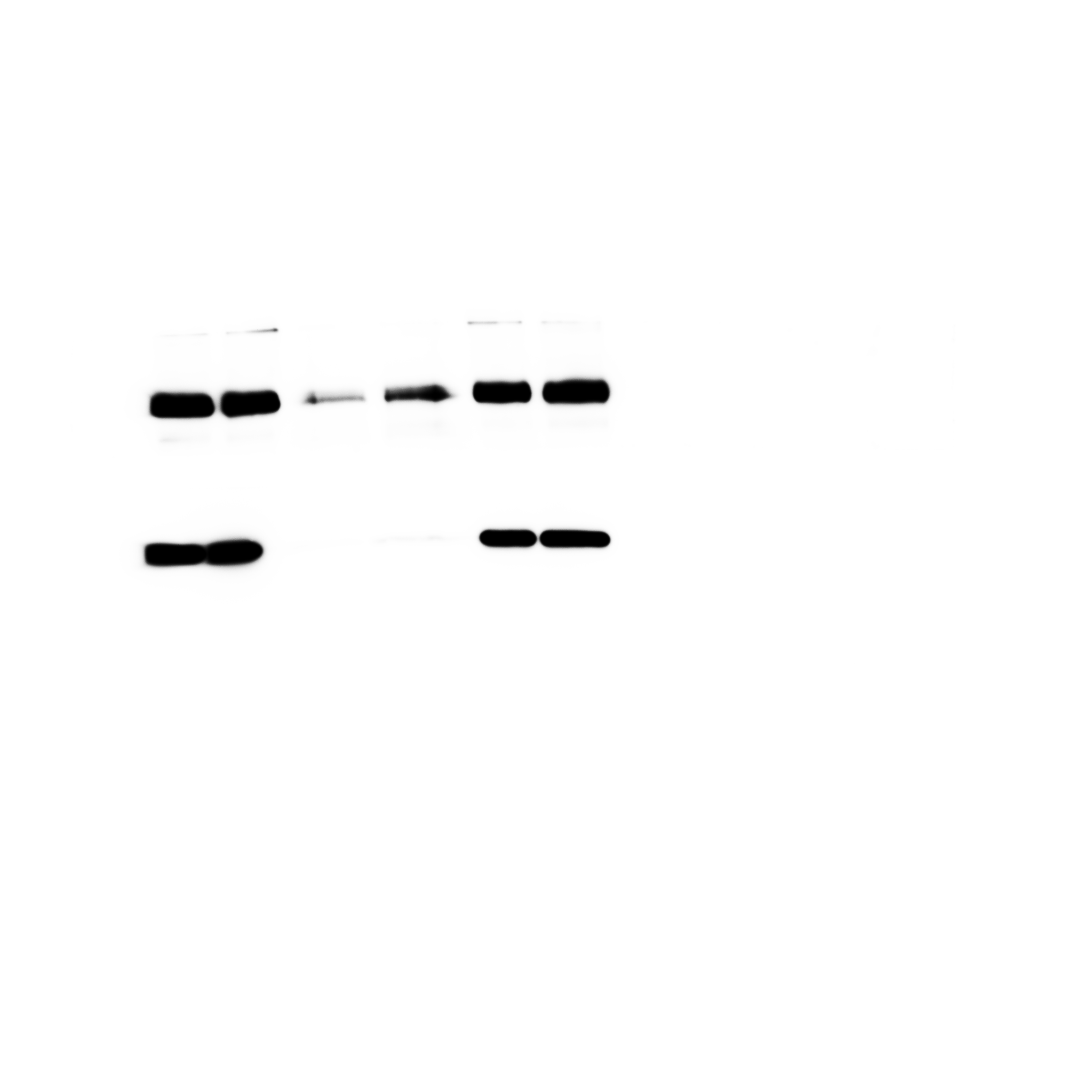

Supplement: Figure 4—source data 1. [file elife-83545-fig4-data1.zip › Figure 4/Fig_4C_synapsin_original.tiff]

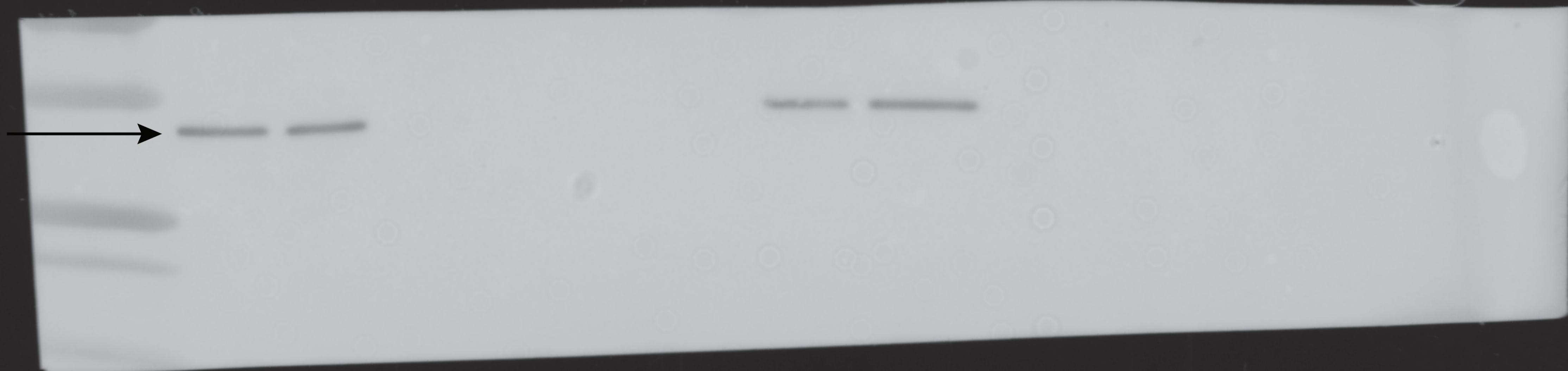

Supplement: Figure 4—source data 1. [file elife-83545-fig4-data1.zip › Figure 4/Fig_4C_syntaxin_input_annotated.pdf]

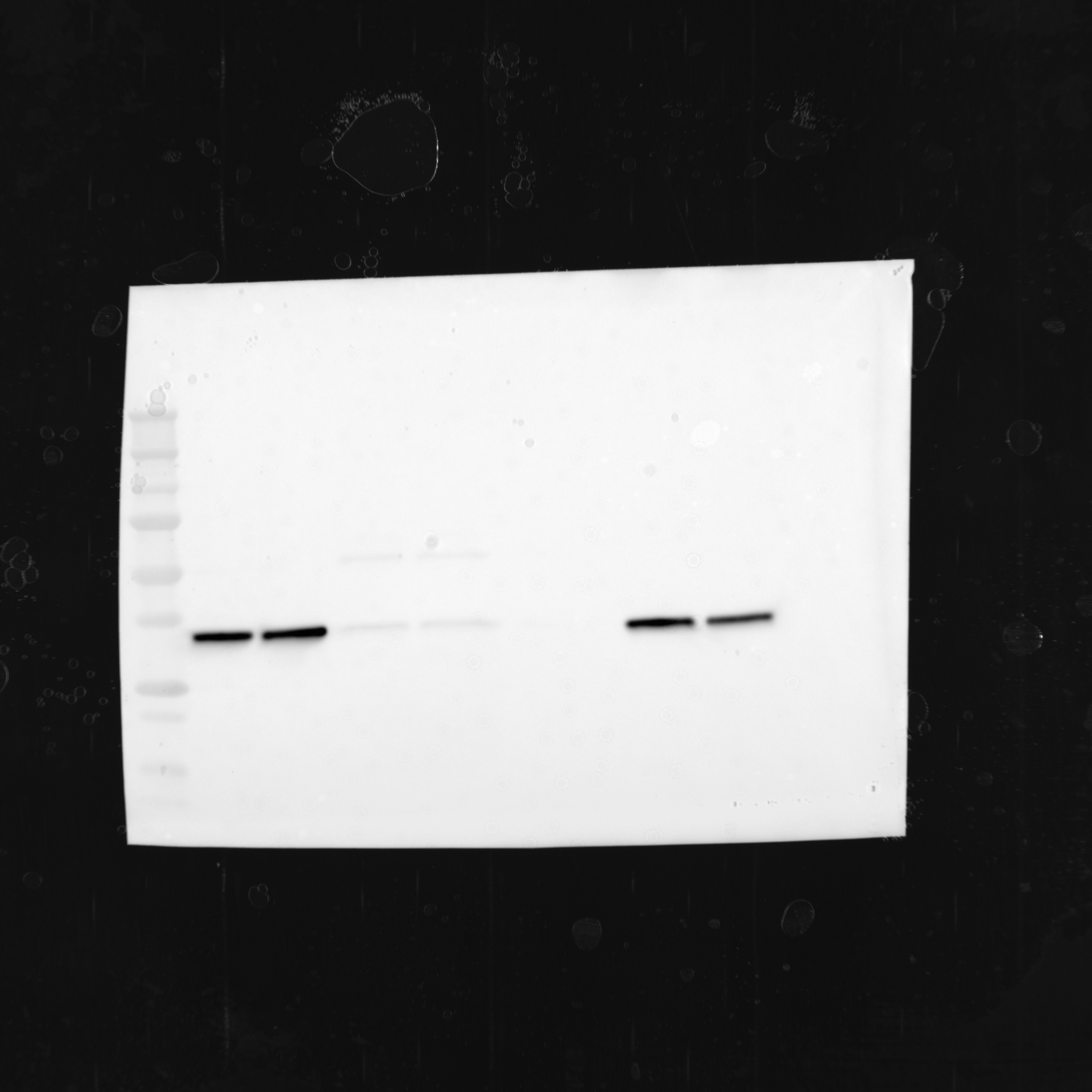

Supplement: Figure 4—source data 1. [file elife-83545-fig4-data1.zip › Figure 4/Fig_4F_syntaxin_original.tiff]

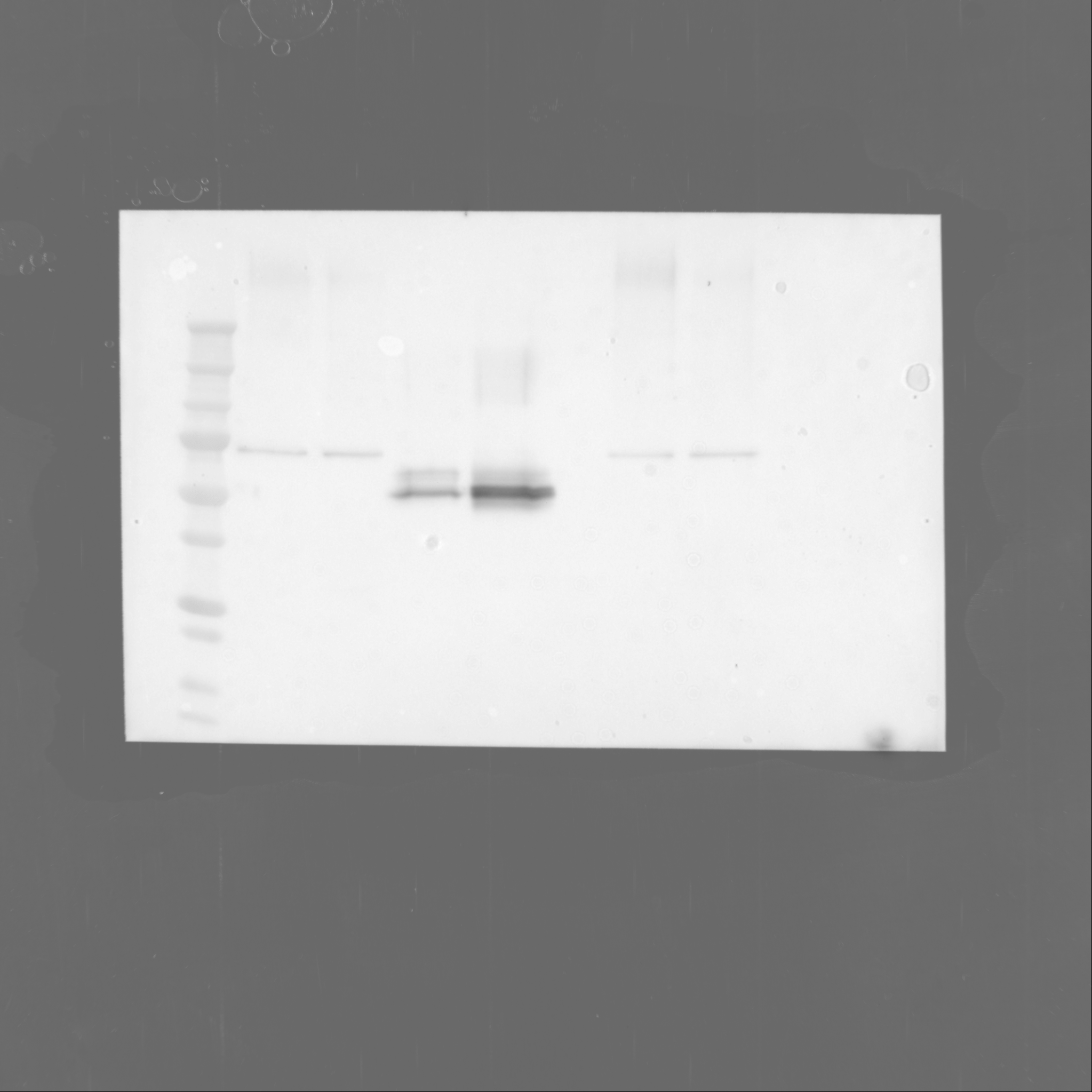

Supplement: Figure 4—source data 1. [file elife-83545-fig4-data1.zip › Figure 4/Fig_4F_draper_original.tif]

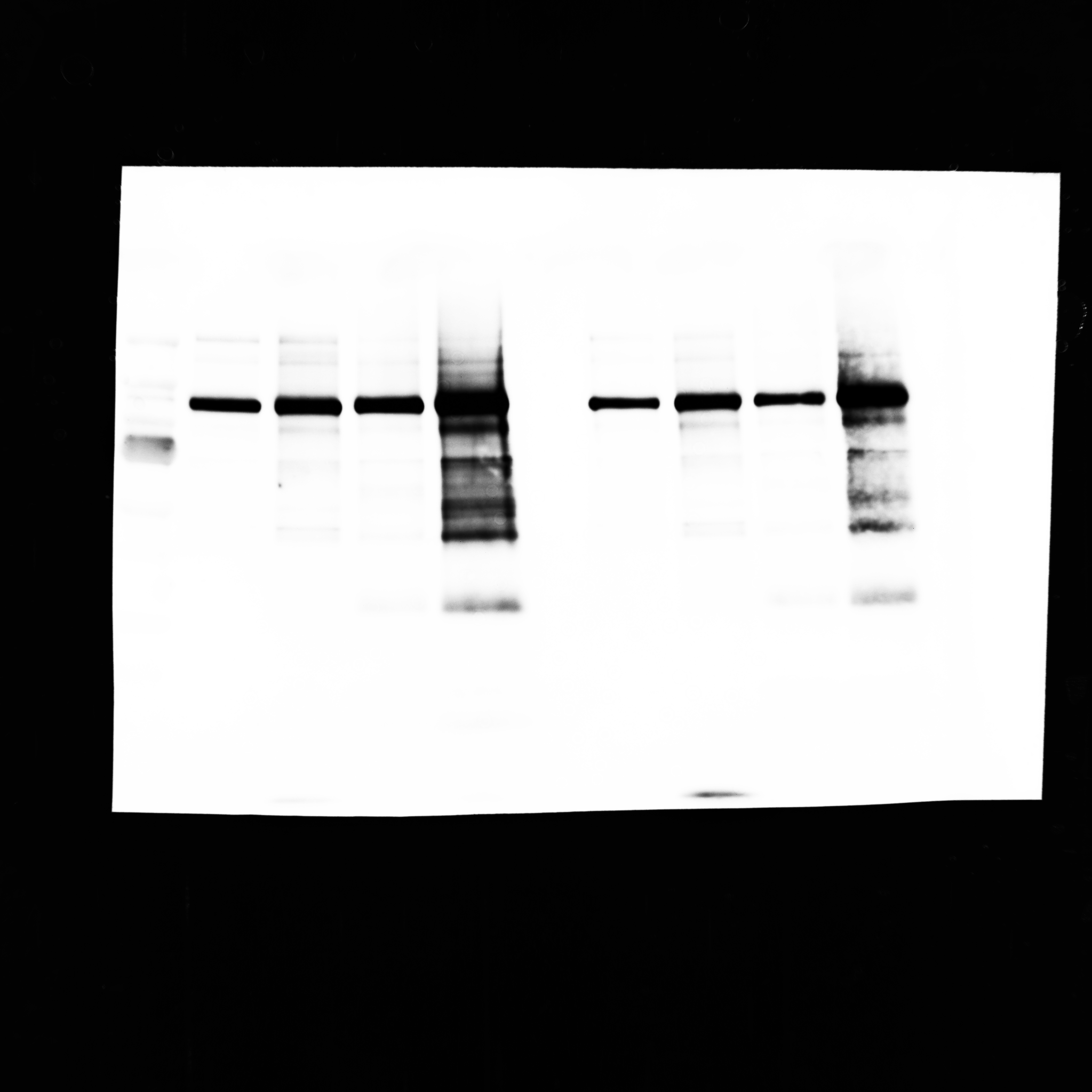

Supplement: Figure 4—source data 1. [file elife-83545-fig4-data1.zip › Figure 4/Fig_4B_biotin_original.tif]

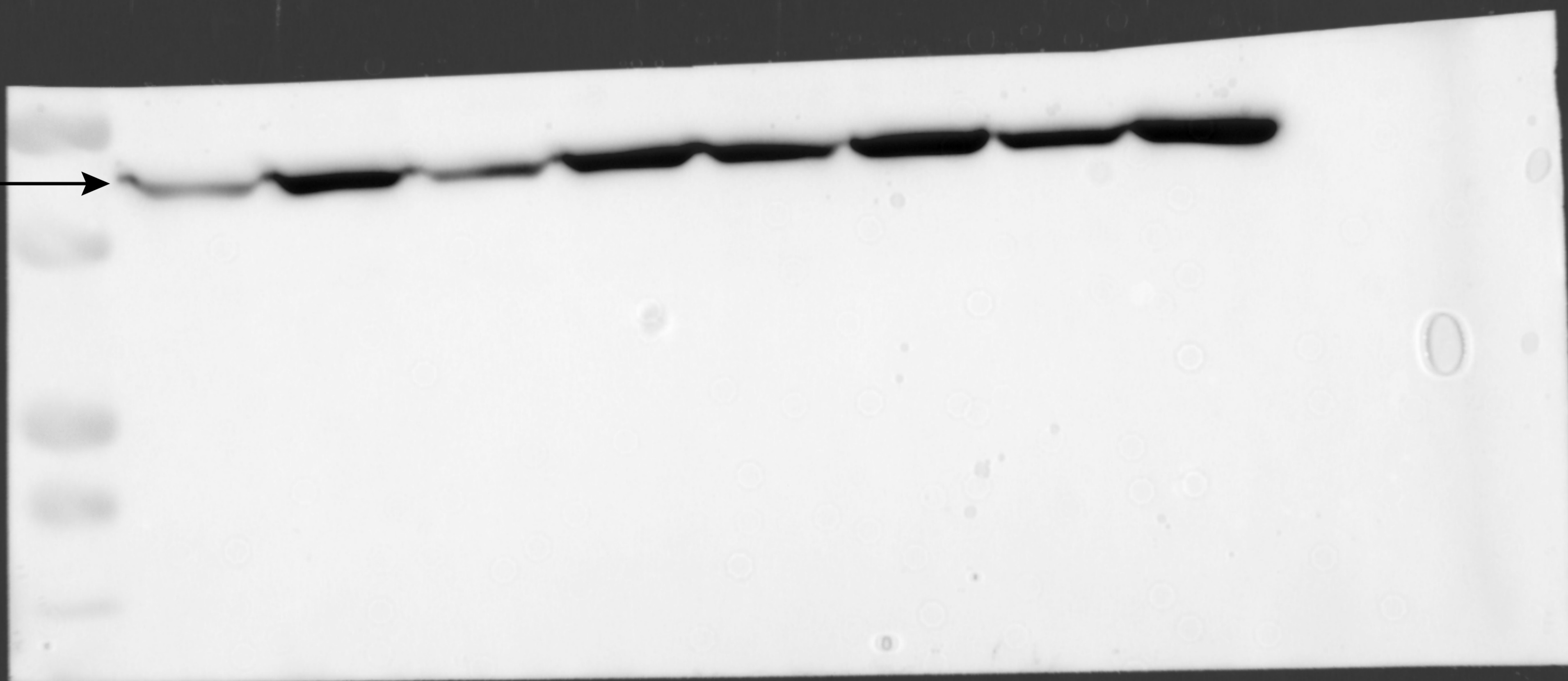

Supplement: Figure 6—source data 1. [file elife-83545-fig6-data1.zip › Figure 6/Fig_6D_actin_annotated.pdf]

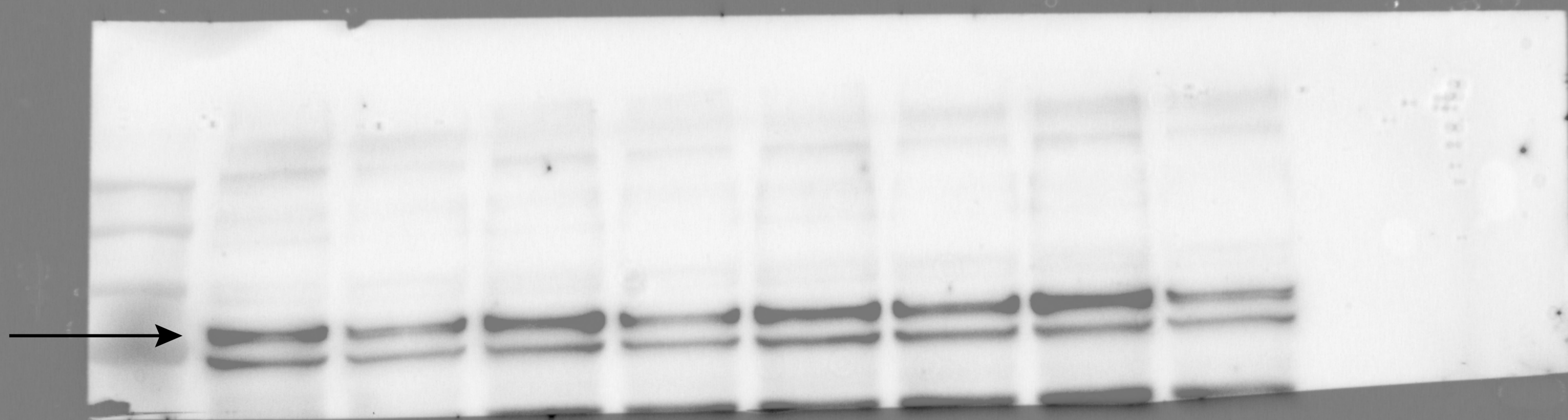

Supplement: Figure 6—source data 1. [file elife-83545-fig6-data1.zip › Figure 6/Fig_6D_FLAG_annotated.pdf]

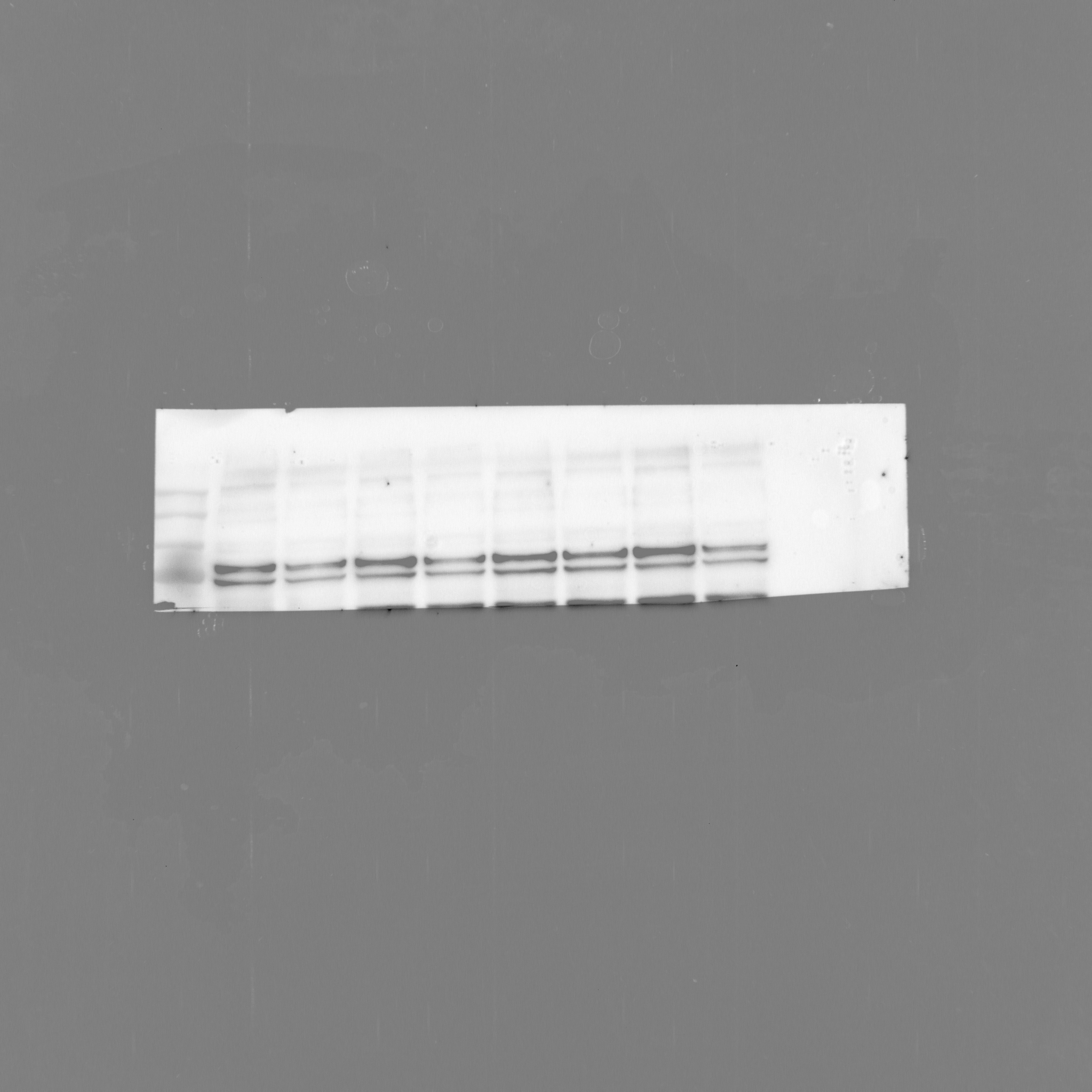

Supplement: Figure 6—source data 1. [file elife-83545-fig6-data1.zip › Figure 6/Fig_6D_FLAG_original.tiff]

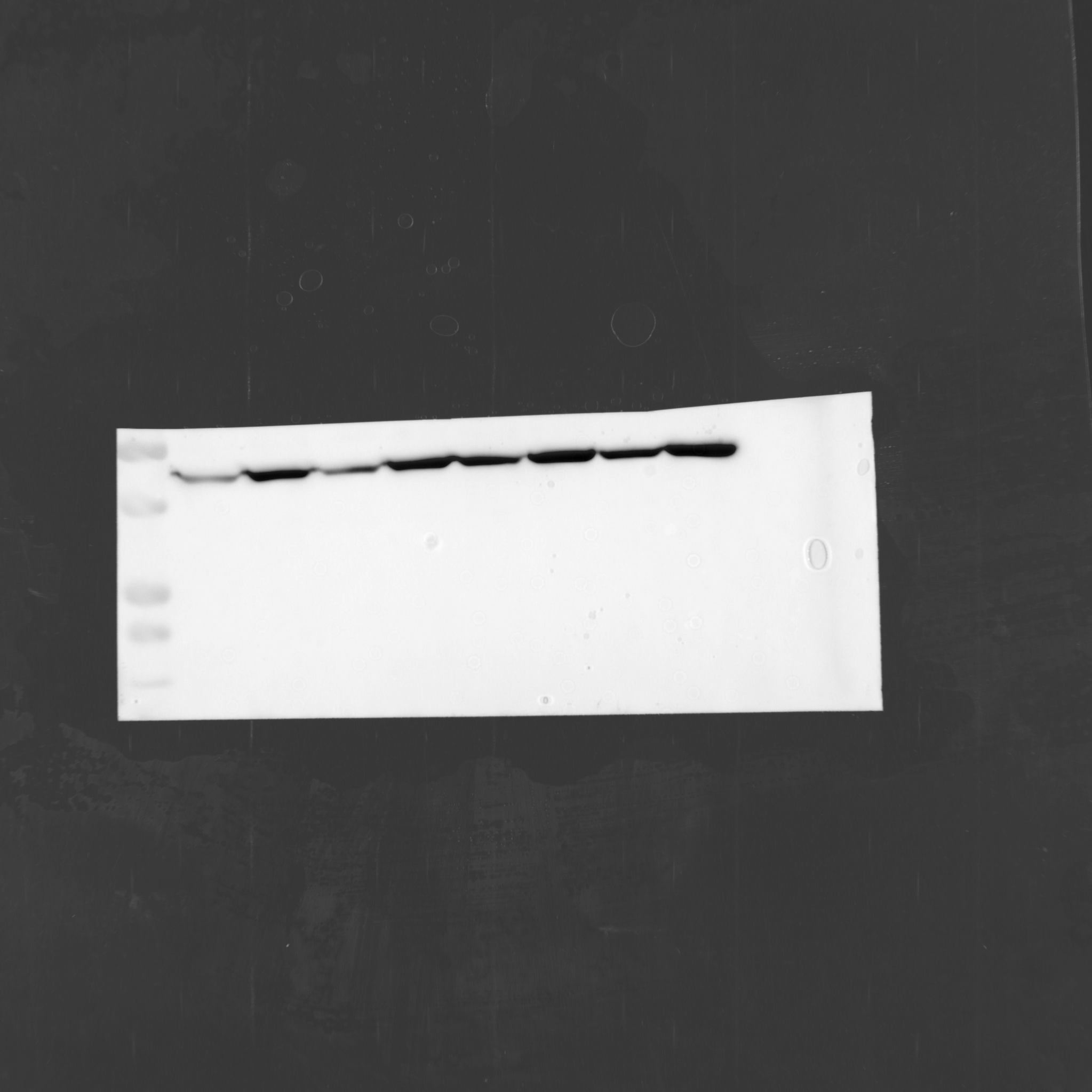

Supplement: Figure 6—source data 1. [file elife-83545-fig6-data1.zip › Figure 6/Fig_6D_actin_original.tiff]
